# Dosing practices, pharmacokinetics, and effectiveness of allopurinol in gout patients receiving dialysis: a scoping review

**DOI:** 10.1007/s40620-025-02269-7

**Published:** 2025-03-25

**Authors:** Noha A. Kamel, Michael A. Stokes, Daniel F. B. Wright, Kamal Sud, Surjit Tarafdar, Ronald L. Castelino, Sophie L. Stocker

**Affiliations:** 1 School of Pharmacy, Faculty of Medicine and Health, The University of Sydney, A15 Pharmacy and Bank Building, Science Road, Camperdown, NSW 2006 Australia; 2 Department of Clinical Pharmacy and Pharmacy Practice, Faculty of Pharmacy, Mansoura University, Mansoura, 35516 Egypt; 3 Paediatric Intensive Care Unit, Department of Pharmacy, The Children’s Hospital at Westmead, Sydney, NSW 2031 Australia; 4 Department of Clinical Pharmacology and Toxicology, St Vincent’s Hospital, Sydney, NSW 2010 Australia; 5 Faculty of Medicine, Vincent’s Clinical School, University of New South Wales, Sydney, NSW 2052 Australia; 6 Nepean Clinical School, The University of Sydney, Sydney, NSW 2006 Australia; 7 Department of Renal Medicine, Nepean Kidney Research Centre, Nepean Hospital, Kingswood, NSW 2750 Australia; 8 Department of Nephrology, Blacktown Hospital, Blacktown, Sydney, NSW 2148 Australia; 9 School of Medicine, University of Western Sydney, Sydney, NSW 2751 Australia; 10 Department of Nephrology, Kasturba Medical College, Manipal, Karnataka 576104 India; 11 Pharmacy Department, Blacktown Hospital, Blacktown, Sydney, NSW 2148 Australia; 12 Sydney Musculoskeletal Health, Faculty of Medicine and Health, The University of Sydney, Sydney, NSW 2006 Australia

**Keywords:** Allopurinol, Urate, Dialysis, Pharmacokinetics, Gout

## Abstract

Urate and oxypurinol, allopurinol’s active metabolite, are predominantly eliminated by the kidneys. Therefore, optimising allopurinol dosing in patients on dialysis is challenging. This review explores allopurinol dosing practices, oxypurinol pharmacokinetics, and effectiveness in gout patients receiving haemodialysis or peritoneal dialysis (PD). Five databases and grey literature were searched. Studies on gout patients on allopurinol, receiving dialysis, and reporting dosing, pharmacokinetics, or effectiveness (reduction in urate and/or gout flares) were included. Abstract, full text screening and data extraction were done by two authors. Studies were grouped by dialysis modality. Eighteen studies were identified including 390 patients, most (*n* = 274, 70%) on haemodialysis with allopurinol administered after dialysis. The peritoneal dialytic clearance of oxypurinol (3.14 mL/min, *n* = 5) and urate (2.7–4 mL/min, *n* = 25) was similar. The haemodialytic clearance was 78–137 mL/min for oxypurinol (*n* = 21) and 80–165 mL/min for urate (*n* = 19). Allopurinol doses were higher in haemodialysis (100–600 mg/day) than PD (110–125 mg/day). Haemodialysis sessions decreased oxypurinol and urate concentrations by 39–57% (*n* = 30) and 56–71% (*n* = 6), respectively. Over time (1–230 days), urate concentrations in haemodialysis (*n* = 85) reduced by 14–41%. Target serum urate (< 0.36 mmol/L) was achieved in 61% (20/33) and 47% (13/28) of haemodialysis and PD patients, respectively. Gout flares decreased from 2 to 0.1 attacks/year in patients receiving dialysis (*n* = 79). Oxypurinol and urate clearance by haemodialysis was higher than PD, necessitating higher doses of allopurinol. POST dialysis allopurinol doses titrated to target urate are suggested. Future studies considering the impact of dialysis modality on allopurinol dose requirements are needed.

**Graphical abstract:**

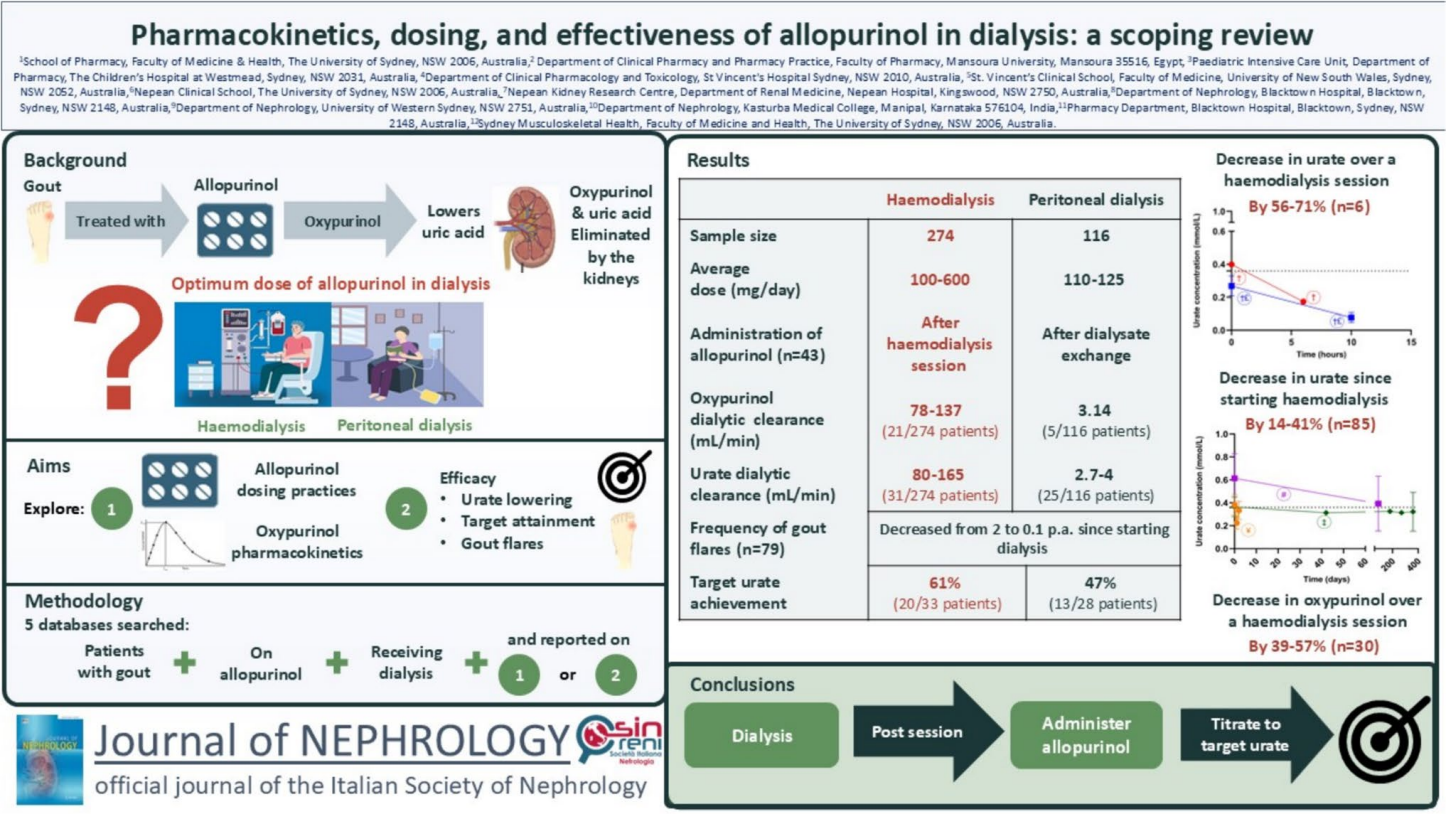

**Supplementary Information:**

The online version contains supplementary material available at 10.1007/s40620-025-02269-7.

## Introduction

Gout is the most common inflammatory arthritis in men worldwide [1, 2]. Chronically elevated serum urate concentrations (hyperuricaemia) contribute to the deposition of monosodium urate (MSU) crystals in joints [3]. These MSU crystals trigger an inflammatory response leading to painful acute gout flares [4]. Treatment and prevention of these gout flares are the cornerstones of gout management. The acute gout flares are treated using non-steroidal anti-inflammatory drugs (NSAIDs), corticosteroids, or colchicine, whereas chronic management to prevent gout flares involves reducing serum urate concentrations with urate-lowering therapies [5].

Allopurinol, a xanthine oxidase inhibitor, is the first-line urate-lowering therapy [6]. It prevents the synthesis of uric acid thereby reducing serum urate concentrations [6]. Allopurinol is a prodrug, and its active metabolite oxypurinol is responsible for much of its clinical effect [7, 8]. The optimal dose of allopurinol is selected using a treat-to-target approach. This approach involves starting with a low dose (50–100 mg based on the estimated glomerular filtration rate (eGFR) in patients with impaired kidney function [9]) and then gradually titrating the dose until target serum urate concentrations (0.36 mmol/L, or 0.3 mmol/L in presence of tophi) are achieved [6, 9].

The use of allopurinol among people with kidney failure on dialysis is common as the risk of gout increases as kidney function decreases. Indeed, the prevalence of gout is threefold higher in people with severely reduced GFR and kidney failure (GFR categories G4–G5) compared to those with early (GFR categories G1–G2) stages of chronic kidney disease [10–12]. Since oxypurinol is predominantly cleared by the kidneys [7], known to be dialysable [13, 14], and dialysis itself is also known to eliminate urate [15], optimal dosing of allopurinol to achieve target serum urate concentrations in people receiving dialysis can be challenging. In addition, the various dialysis modalities, as well as blood flow and dialysate flow rates can impact oxypurinol and urate elimination differently [15]. Due to limited evidence, there are very few guidelines to support allopurinol prescribing decisions in gout patients receiving dialysis (Table S1, Online resource 1). Since a comprehensive understanding of the pharmacokinetics and effectiveness of oxypurinol in people with gout receiving dialysis is required to guide optimal dosing, this scoping review was conducted to explore allopurinol dosing practices, the pharmacokinetics of oxypurinol, and the effectiveness of allopurinol (i.e., urate lowering and frequency of gout flares) in patients with gout receiving dialysis.

## Methodology

This scoping review was conducted in accordance with the Preferred Reporting Items for Systematic Reviews and Meta-analyses extension for scoping reviews checklist (PRISMA-SCR) [16].

### Eligibility criteria

All studies conducted in patients with gout who were taking allopurinol, receiving haemodialysis or peritoneal dialysis (PD), and reporting on the pharmacokinetics or efficacy of allopurinol were included. Studies were limited to human data. There were no restrictions on the language or year of publication. As for study design, case reports, case series, and short communications were included, while reviews, editorials, abstracts and protocols were excluded. The reference lists of relevant publications (even those excluded) were mined for studies of interest.

### Information sources and search strategy

Five databases were searched including: Ovid MEDLINE, Ovid Embase, EBSCOhost CINAHL, Scopus, and Web of Science from inception of the databases till October 19th, 2023. The search was updated on May 1st, 2024. As for grey literature, clinical trial registries (clinicaltrials.gov, World Health Organization International Clinical Trials Registry Platform, and Cochrane Central Register of Controlled Trials) were searched for ongoing trials evaluating the use of allopurinol in dialysis. The search keywords and strategies were developed with the assistance of a librarian and included ‘allopurinol’, ‘antigout agent’, ‘dialysis’, ‘oxypurinol’, ‘pharmacokinetic’ and ‘uric acid’. Respective database-specific vocabulary (e.g. Medical Subject Headings) were used where permitted in databases such as ‘allopurinol’, ‘dialysis’, ‘pharmacokinetics’, ‘uric acid’. The search strategy for all five databases and clinical trial registries are provided in Online resource 2.

### Selection process

Studies identified from the search strategy were imported into Covidence (Veritas Health Innovation, Melbourne, Australia) where duplicates were removed both automatically and manually by one of the authors (NK). Following the duplicate removal process, title, and abstract screening was carried out independently by two reviewers (NK and MS). Full texts of the eligible studies were reviewed independently by the same two reviewers. Any conflicts during the screening or review processes were resolved through consensus with a third reviewer (SS) whenever necessary. If required, translation of studies into English was conducted.

### Data extraction process

An electronic data extraction form was developed (Excel) collaboratively by two authors (NK and MS). Data extraction was performed by one reviewer (NK) and the accuracy of data input confirmed by another (MS). The outcomes of interest were the dose regimen of allopurinol administered, pharmacokinetics of oxypurinol and efficacy of allopurinol (including attainment of target urate concentrations and frequency of gout flares). The following data were collected:

- Study characteristics including location of the study, number of study sites, sample size and study design.
- Demographics of the study population (age, gender, and ethnicity) including gout diagnosis (definition or method of diagnosis).
- Duration of dialysis, dialysis type and details of dialysis mode including dialyser type, dialysis adequacy, blood and dialysate flow rates, dialysis flux, dialysis dose, frequency, and duration of dialysis sessions in haemodialysis, technique, peritoneal transport status, dialysis regimen, dwell frequency and duration and dialysis adequacy in peritoneal dialysis.
- Duration of allopurinol therapy, dose regimen (dose and dose interval) of allopurinol including time of administration in relation to the haemodialysis session or peritoneal dialysis dwell and average daily dose.
- Pharmacokinetics of oxypurinol including dialytic clearance, and plasma concentrations (relative to dialysis session and/or commencement of dialysis).
- Efficacy of allopurinol including serum urate concentrations (relative to dialysis session and/or commencement of dialysis), attainment of target urate concentrations and frequency of gout flares.

For population pharmacokinetic models the following additional information was collected: the model selection criteria, model evaluation methods, model structure including estimates of fixed effect parameters, random effects estimates (between subject variability, covariance etc.), covariate models included in the final model and ETA and EPISILON shrinkage.

Authors of the included studies were contacted (multiple attempts) in case of missing or unclear information. If the authors were unreachable, the information was deemed as unavailable. In line with the objectives of this scoping review and the Joanna Briggs institute guidance for conducting scoping reviews [17], critical appraisal of the included studies was not undertaken.

### Synthesis and data analysis

Studies were grouped by dialysis modality (haemodialysis or peritoneal dialysis). Dialysis duration was defined as the time on dialysis (days, months, or years) since study enrolment. Duration of allopurinol therapy was defined as the time on allopurinol since study enrolment. Oxypurinol and urate concentrations obtained before or after commencement of dialysis/dialysis session or dwell were referred to as “PRE” or “POST” concentrations, respectively. If the mean and standard deviation of concentrations were not reported, this was calculated from the median and range (http://vassarstats.net/median_range.html) based on the publication of Hozo et al*.* [18]. Data depicted only in figures were digitised using WebPlot Digitiser software [19]. To enable comparison across studies, oxypurinol and urate concentrations were standardised to SI units (mmol/L) and the time since commencement of dialysis to days. The average daily dose of allopurinol was calculated using the dose and dosing frequency data. For venous oxypurinol or urate concentrations, the change in concentrations was calculated as follows:

$${\text{Change}}\;{\text{in}}\;{\text{concentration}}\;{\text{over}}\;{\text{a}}\;{\text{dialysis session}} = {\text{first}}\;{\text{available}}\;{\text{POST}}\;{\text{session}}\;{\text{concentration}} - {\text{last}}\;{\text{available}}\;{\text{PRE}}\;{\text{session}}\;{\text{concentration}}$$

Or $${\text{mean POST session concentration}} - {\text{mean PRE session concentration}}$$

$${\text{Change}}\;{\text{in}}\;{\text{concentration}}\;{\text{since}}\;{\text{starting}}\;{\text{dialysis}} = {\text{first}}\;{\text{available}}\;{\text{POST}}\;{\text{dialysis}}\;{\text{start}}\;{\text{concentration}} - {\text{last}}\;{\text{available}}\;{\text{PRE}}\;{\text{dialysis}}\;{\text{start}}\;{\text{concentration}}$$

Or $${\text{mean POST dialysis start concentration}} - {\text{mean PRE dialysis start concentration}}$$

The haemodialytic clearance was calculated using the mean PRE dialysis session concentration, mean POST dialysis session concentration, and blood flow rate using the A-V method [20, 21].

$${\text{Haemodialytic}}\;{\text{clearance}} = \left( {\frac{{{\text{PRE}}\;{\text{session}}\;{\text{concentration}} - {\text{POST}}\;{\text{session}}\;{\text{concentration}}}}{{{\text{PRE}}\;{\text{session}}\;{\text{concentration}}}}} \right) \times {\text{blood}}\;{\text{flow}}\;{\text{rate}}.$$

In peritoneal dialysis, the mean dialytic clearance of oxypurinol or urate was calculated from the individual or median (range) clearance normalised to body surface area as

$${\text{Mean}}\;{\text{dialytic}}\;{\text{clearance}}\left( {{\text{mL/min}}{.}} \right) = \sum {\text{of}}\;{\text{individual}}\;{\text{patients}}\;{\text{clearances}}\div{\text{number of patients}}$$ or $${\text{Mean}}\;{\text{dialytic}}\;{\text{clearance}}\left( {{\text{mL}}/{\text{min}}.} \right) = ({\text{Clearance (mL/min./1.73 m2)}}\times{\text{BSA (m2)}})/1.73$$.

The mean renal clearances were calculated in the same way, then total clearance was obtained by summing the mean peritoneal and renal clearances.

The number (percentage) of patients with > 25% of their urate concentrations below the target for each dialysis modality was digitized from a figure in Yeo et al*.*’s study [22].

## Results

### Study selection

**Fig. 1:**
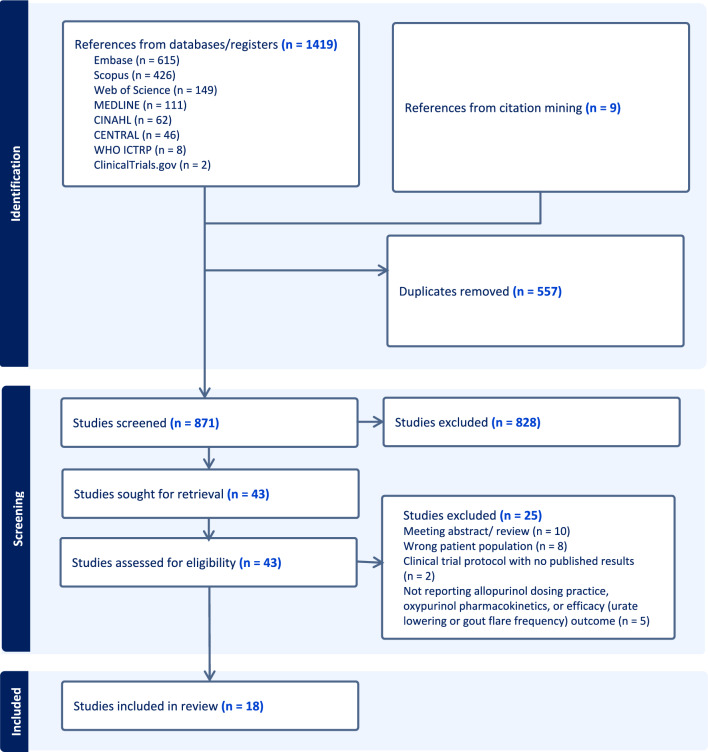
PRISMA 2020 flow diagram for scoping reviews

Overall, 1428 records were identified from the databases and through citation mining. Following the duplicate removal process, 871 records were screened based on the study title and abstract. Of these, 43 studies qualified for full text review with 18 studies remaining eligible for data extraction and analysis (Fig. 1). Twenty-five articles were excluded from full text review because (i) they were not conducted in people with gout (*n* = 3), (ii) patients were not given allopurinol (*n* = 2), (iii) patients were not receiving dialysis (*n* = 3), (iv) the study did not report the outcomes of interest (*n* = 5), (v) the study design was ineligible (*n* = 10). Two clinical trials (*n* = 2) were identified but not included in the analysis. One trial was already complete with the full text article [23] already included, while the results for the other trial (NCT02477488) were unavailable (online resource 2).

### Study characteristics

Most studies were observational (both retrospective and prospective), conducted in the United States, each at a single centre, with sample sizes ranging from 1 to 158 patients (Table 1). Some (10/18) studies were conducted only in gout patients while others (8/18) also included patients with asymptomatic hyperuricaemia (Table 1). Overall, data from 390 patients (274 patients (70%) receiving haemodialysis and 116 patients (30%) receiving peritoneal dialysis) were available. Patients were predominantly male (84 male/41 female) and of White/European (Caucasian) ethnicity (75/188 patients). Five studies did not report the gender of allopurinol users [8, 24–27] and eight studies did not report their ethnicity [8, 13, 24–29]. No studies were conducted in patients undergoing continuous renal replacement therapy (CRRT) or home haemodialysis. Heterogeneity between studies was observed with respect to allopurinol dosing regimens, duration of dialysis sessions, and reporting of oxypurinol pharmacokinetics and effectiveness. Hence, a narrative synthesis approach was adopted.

**Table 1:** Characteristics of tudies investigating the pharmacokinetics and pharmacodynamics of allopurinol in patients on dialysis

Study (*n* = 18)	Country	No. of centres	Sample size	Age (years)	Male (%)
Haemodialysis studies					
Alkilany et al*.,* 2022 [64]	USA	Single	10^a^	64 ± 15	60
Arenas et al*.,* 2021 [24]	Spain	Single	17^b^	66.5 ± 13.8	65
Elion et al*.,* 1980 [27]	USA	Single	3^b^	NR	NR
Hande et al*.*, 1984 [8]	USA	Single	13^b^	NR	NR
Hayes et al*.,* 1965 [37]	USA	Single	2^b^	37 and 38	100
Hsu et al*.,* 2004 [34]^c^	Taiwan	Single	1	43	100
Johnson et al*.*, 1979 [36]^c^	USA	Single	1	46	100
Matsuda et al*.*, 1993 [35]^c^	Japan	Single	1	44	100
Reiter et al*.,* 1998 [29]^c^	Germany	Single	1	53	100
Richard O. Day et al*.,* 2012 [13]^c^	Australia	Single	1	49	100
Rohn et al*.,* 2020 [26]	Germany	Multisite	186^b^	NR	NR
Rutherford et al*.,* 2021 [32]	Scotland	Multisite	28^b^	56.5 ± 11.3	44
Shelmadine et al*.,* 2009 [28]	USA	Single	12	45.8 ± 13.6	66
Wright et al*.,* 2017 [33]	New Zealand	Single	6	63 [28–72]^d^	83
Peritoneal dialysis studies					
Diez et al*.*, 2021 [25]	Spain	Single	8^b^	53.5 [42.3–63.3]^e^	65
Wilson et al*.,* 2024 [23]	New Zealand	Multisite	5	65 ± 17^f^	80
Haemodialysis and peritoneal dialysis studies					
Ohno et al*.,* 2005 [38]	Japan	Multisite	158^b^	NR	NR
Yeo et al*.,* 2019 [22]	New Zealand	Single	42^g^	61 ± 14.4	75

Data represented as mean ± SD unless otherwise specified

*NR* not reported

^a^Gout patients with available serum urate concentrations PRE and POST haemodialysis (relevant group)

^b^Number of patients on allopurinol within a mixed cohort of gout/hyperuricemic patients

^c^Case report

^d^Median [range]

^e^Median [inter-quartile range]

^f^Calculated mean ± SD age for the 5 patients

^g^Patients with gout and on allopurinol

### Dialysis conditions

A range of dialysers were used across the studies with dialysis conditions infrequently reported in detail (Table 2). For haemodialysis, blood flow rates ranged from 200 to 400 mL/min and the dialysate flow rates ranged from 160 to 800 mL/min. The duration of dialysis sessions ranged from 3 to 8 h. Dialysis adequacy (measured by Kt/*V*, where *K* = urea clearance by dialyser (L/min), *t* = time (min), *V* = volume of body fluids (L)) was only reported in two studies in haemodialysis with most (47/55, 85%) patients receiving a Kt/V > 1.3 [24, 28], in line with guideline recommendations for Kt/V of 1.4 for a dialysis session [30]. The Kt/V representing both the residual kidney function and peritoneal dialysis components ranged from 1.9 to 2.3 for 20 patients receiving peritoneal dialysis [25], consistent with guideline recommendations for a total Kt/V not below 1.7 [31]. The peritoneal transport status (an indication of peritoneal membrane permeability) of the patients in this study was reported as high-average to high (70%), high (20%), or low-average to low (10%) [25]. According to the preliminary data of the study, peritoneal dialytic clearance was not significantly different among patients with different peritoneal transport status.

**Table 2:** Haemodialysis modality details for patients on dialysis receiving allopurinol

Study (*n* = 15)	Dialyser	Blood flow rate (mL/min)	Dialysate flow rate (mL/min)	Flux	Session duration (hours)	Duration of dialysis	Dialysis frequency
Alkilany et al*.,* 2022 [64]	NR	NR	NR	NR	NR	1–4 years	NR
Arenas et al*.,* 2021 [24]	NR	< 400 (22%), > 400 (72%)	500 or 800	High efficacy 64.5%	NR	4 months	NR
Elion et al*.,* 1980 [27]	NR	NR	NR	NR	6	4–26 days	Twice weekly
Hande et al*.,* 1984 [8]	Gambro Lundia Major dialyzer	200	NR	NR	4	4 h	NR
Hayes et al*.,* 1965 [37]	2-layer modified Kiil dialyzer	250	NR	NR	8	3 months	Twice weekly
Hsu et al*.*, 2004 [34]^a^	NR	NR	NR^b^	NR	NR	11 months	NR
Johnson et al*.,* 1979 [36]^a^	Mini Kiil, then Gambro, then Cordis Dow 5	NR	NR	NR	7–8 h, then 6–7 h, then 4 h sessions	7 years	Thrice weekly
Matsuda et al*.*, 1993 [35]^a^	NR	NR	NR	NR	NR	15 months	Thrice weekly
Reiter et al*.,* 1998 [29]^a^	NR	NR	NR	NR	3	32 months	Thrice weekly
Richard O. Day et al*.,* 2012 [13]^a^	NR	NR	NR	NR	4	1 year	Thrice weekly
Rohn et al*.,* 2020 [26]	NR	NR	NR	NR	NR	34 (3–100)^c^ months	NR
Rutherford et al*.,* 2021 [32]	Fistula or graft (92.9%), line (7.1%^d^)	NR	NR	NR	NR	30 (15–61)^e^ months	Thrice weekly
Shelmadine et al*.,* 2009 [28]	Polysulfone membranes (95% Fresenius-160, 5% Fresenius-180)	376.49	800	NR	4	3 months	Thrice weekly
Wright et al.*,* 2017 [33]	Fresenius FX 80, (*n* = 1) or Hemoflow F8HPS polysulfone (*n* = 5)	200 (low-flux, 83%), 300 (high-flux, 17%)	530 (160–700)^c^	Low-flux filter (n = 5), High-flux (n = 1)	5	48 h	Thrice weekly
Yeo et al*.,* 2019 [22]	NR	NR	NR	NR	5 (79%)	≥ 3 months	HD: (58%) Thrice weekly

Data reported as mean ± SD unless specified otherwise

*NR* not reported, *HD* haemodialysis

^a^Case report

^b^Only reported maintenance HD with low calcium dialysate, calcium concentration of 1.25 mEq/L, calcium efflux about 500–700 mg per session

^c^Median (range)

^d^Dialysis access

^e^Median (interquartile range)

### Allopurinol dosing regimen

Only eight studies (*n* = 59) reported the time of allopurinol administration in relation to the dialysis session (haemodialysis) or dwell (peritoneal dialysis) (Table 3). Allopurinol was most commonly (43/58 patients) administered after the haemodialysis session or in peritoneal dialysis when dialysate exchange was completed. Less than a quarter (10/47) of patients were on allopurinol for ≥ 1 year prior to commencing dialysis [13, 23, 27, 29, 32–36]. Higher average daily allopurinol doses were administered in haemodialysis; 100–600 mg/day (dose range 100–1000 mg) than peritoneal dialysis; 110–125 mg/day (dose range 50–200 mg) (Table 3).

**Table 3:** Allopurinol dosing details for patients on dialysis receiving allopurinol

Study (*n* = 18)	Sample size	Allopurinol maintenance dose (mg)	Frequency	Average maintenance dose (mg/day)	Time administered relative to dialysis	Duration of allopurinol therapy
Haemodialysis studies						
Alkilany et al*.,* 2022 [64]	10^a^	NR	NR	NR	NR	1–10 years
Arenas et al*.,* 2021 [24]	17^b^	NR	NR	NR	NR	7.1 ± 7.2 years
Elion et al*.,* 1980 [27]	3^b^	400	Every 4th day	114.3	POST session	4 days
Hande et al*.,* 1984 [8]	13^b^	300–600	NR	NR	PRE^c^ session	24 h
Hayes et al*.,* 1965 [37]	2^b^	600–800^d^	3 doses^e^	304.3	PRE^e^ session	≈ 2 months
Hsu et al*.*, 2004 [34]^f^	1	200	Daily	200	NR	11 months
Johnson et al*.,* 1979 [36]^f^	1	400 then 200	Daily	409	NR	8 years
Matsuda et al*.*, 1993 [35]^f^	1	300	Daily	300	NR	39 months
Reiter et al*.,* 1998 [29]^f^	1	200–400 then 1000	Daily	240	PRE except 600 mg POST session	3 years
Day et al*.,* 2012 [13]^f^	1	250, then 300, then 350, then 200	Daily	239	POST session	3 years
Rohn et al*.,* 2020 [26]	186^b^	NR	NR	NR	NR	Any time after dialysis start till end point of the study^g^
Rutherford et al*.,* 2021 [32]	28^b^	300	Thrice weekly	130.4	POST session	12 months
Shelmadine et al*.,* 2009 [28]	12	300	bid	600	NR	3 months
Wright et al*.,* 2017 [33]	6	100	Daily	100	POST session	48 h
Peritoneal dialysis studies						
Diez et al*.*, 2021 [25]	8^b^	100–150^h^	Daily	125	PRE^i^	1 month
Wilson et al*.*, 2024 [23]	5	50–200	Daily	125	POST dwell 1	24 h
Haemodialysis and peritoneal dialysis studies						
Ohno et al*.,* 2005 [38]	158^b^	NR	NR	NR	NR	≥ 2y
Yeo et al*.,* 2019 [22]	42^j^	HD: 121.4 ± 61.6^k^, PD:110 ± 71.8^k^	Daily	HD: 121.4, PD: 110	NR	0.25– > 10 y

Data reported as mean ± SD unless specified otherwise

*NR* not reported, *POST* after single or multiple dialysis sessions or dwells, *PRE* before the dialysis session or dialysis start, *bid* twice daily, *HD* haemodialysis, *PD* peritoneal dialysis

^a^Gout patients with available serum urate concentrations PRE and POST haemodialysis (relevant group)

^b^Number of patients on allopurinol within a mixed cohort of gout/hyperuricemic patients

^c^By 8–10 h

^d^As single oral doses

^e^Immediately prior to 3 separate hemodialyses for both patients to determine oxypurinol and urate dialysance, while patient T.H on non-dialysis days 400 mg/day for 5 days/week, omitted on dialysis days

^f^Case report

^g^Follow up ranged from 3 to 100 months

^h^For 10/20 patients on allopurinol when peritoneal dialysis was started

^i^Patients were on allopurinol when peritoneal dialysis was started, however, the administration time of allopurinol relative to the peritoneal dialysis dwell was not reported

^j^Patients with gout and on allopurinol

^k^Doses provided for 22 HD patients and 20 PD patients

### Oxypurinol pharmacokinetics in dialysis

#### Haemodialysis

Overall, 165 oxypurinol concentrations (94 plasma and 71 serum samples) were available from 26 patients receiving haemodialysis. Oxypurinol concentrations decreased between 39 and 57% after a haemodialysis session (Table 4). The dialytic clearance of oxypurinol ranged from approximately 78–137 mL/min (*n* = 21) (Table 4). The inter-dialytic clearance of oxypurinol was 20 mL/min normalized to a fat-free mass of 70 kg and creatinine clearance of 6 L/h [33]. On two separate occasions, oxypurinol concentrations in the dialysate were reported as 0.112 and 0.191 mmol/L for one patient on an average daily allopurinol dose of 304.3 mg/day (dose range 600–800 mg before session) [37]. There was only one published population pharmacokinetic model of oxypurinol, which is summarised in Table S2, Online resource 1.

**Table 4:** Clearance of oxypurinol in patients on allopurinol receiving dialysis

Study (*n* = 7)	Sample size	Blood sampling times	Plasma oxypurinol concentration (mmol/L)			Oxypurinol dialytic clearance (mL/min)
			PRE dialysis	POST dialysis	Decrease in concentration^a^	
Haemodialysis studies						
Day et al*.,* 2012 [13]^b^	1	PRE and POST session	0.197^c^, 0.162^d^	0.099^c^, 0.064^d^	49.7%, 60.4%	NR
Elion et al*.,* 1980 [27]	3	At 0, 2, 6 h of dialysis session	0.054 ± 0.02^e^	0.027 ± 0.01^e^	46 ± 10%^e^	NR
Hande et al*.,* 1984 [8]	13	PRE and POST session	0.1 ± 0.09^f^	0.062 ± 0.05^f^	39 ± 17%	80.7
Hayes et al*.,* 1965 [37]	2	PRE^g^ and POST^h^ session	0.145 ± 0.039^i^	0.072 ± 0.033^i^	0.073 ± 0.27^j^ (~ 50%)	78 ± 9
Reiter et al*.,* 1998 [29]^b^	1	PRE session	0.148^k^, 0.1^l^	NR	NR	NR
Wright et al*.*, 2017 [33]	6	Hourly during dialysis session, then 24, 24.5, 25, 25.5 26, 28, 30, and 48 h POST dialysis	0.027 ± 0.01^m^	0.011 ± 0.006^m^	56.7%^m^	137 (7)^n^
Peritoneal dialysis studies						
Wilson et al*.*, 2024 [23]	5	0, 2, 4, 6, 8, 10, 24 h after the dose	0.031–0.1354^o^	0.031–0.1349^o^	0.4 ± 0.3% (0–0.9%)^o^	3.14 ± 1.03^p^

Data represented as mean ± SD unless otherwise specified. All concentration units were converted to mmol/L based on the molecular weight of oxypurinol (152.11)

*NR* not reported

^a^The difference between PRE dialysis and POST dialysis oxypurinol concentrations

^b^Case report

^c^On dose 350 mg in 2 different occasions for one patient

^d^On dose 350 mg in 2 different occasions for one patient

^e^Calculated mean ± SD concentrations for the 3 patients at 0 h (PRE), 6 h of dialysis (POST) and percentage of concentration decrease

^f^calculated mean using values digitised from Fig. 5 in the publication

^g^Within 2 h after mid-day allopurinol dose

^h^At 2 h, 8–10 h (4 occasions each)

^i^For patient T.H

^j^Calculated as (mean1-mean2) ± sqrt(SD1 + SD2)

^k^On 200 mg allopurinol given PRE dialysis

^l^On 400 mg allopurinol PRE dialysis plus 600 mg POST dialysis

^m^Values digitised from Fig. 1 in the publication, the change in concentration computed using the PRE and POST concentrations

^n^Population mean (% relative standard error)

^o^Values digitised from Fig. 1 in the publication, PRE refers to the calculated mean ± SD concentration at 4 h after allopurinol dose (by the end of dwell 2), POST refers to the calculated mean ± SD concentration 1 h after dwell 2 dialysate removal and starting dwell 3, then the change in concentration was computed using the PRE and POST concentrations

^p^Calculated mean ± SD dialytic clearance for the 5 patients

#### Peritoneal dialysis

In total, 35 plasma oxypurinol concentrations were available from five patients receiving peritoneal dialysis. The decrease in oxypurinol concentrations after a 4-h dwell of continuous ambulatory peritoneal dialysis (CAPD) was minor (up to 0.9%) for five patients with an average CAPD clearance of 3.14 mL/min [23]. The type or volume of dialysate was not associated with the clearance of oxypurinol for these five patients [23]. The peritoneal dialytic clearance of oxypurinol constituted 64% of the total oxypurinol clearance in CAPD [23]. No studies evaluated the dialytic clearance of oxypurinol in automated peritoneal dialysis.

### Urate control in patients on dialysis receiving allopurinol

**Fig. 2:**
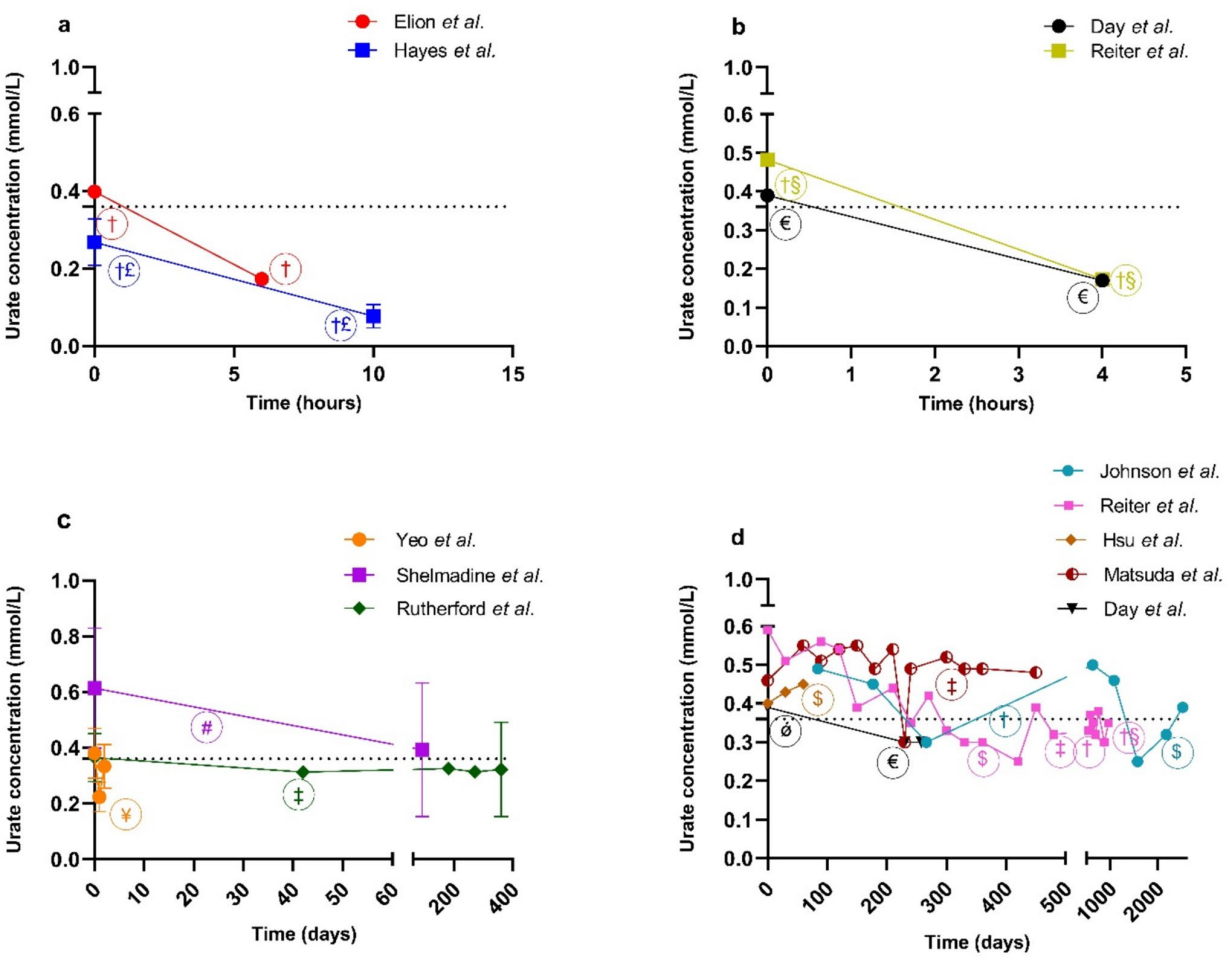
The change in serum urate concentrations in gout patients receiving haemodialysis and allopurinol. The change in serum urate concentrations over a haemodialysis session for a study cohort (**a**) or individual patients (**b**). The change in serum urate concentrations over time (days) since commencement of dialysis for a study cohort (**c**) or individuals (**d**). For study cohorts, the mean ± SD is presented. The doses and frequency of dosing varied across the studies ^†^400 mg allopurinol dose (daily for Johnson et al*.* [36]*,* on non-dialysis days for Hayes et al*.* [37], POST dialysis session for Elion et al*.* [27]); ^£^600–800 mg allopurinol dose PRE dialysis session [37];^†§^400 mg allopurinol PRE dialysis session plus 600 mg POST dialysis session [29]; ^€^250 mg/day allopurinol dose [13]; ^#^600 mg allopurinol dose as 300 mg twice daily [28]; ^‡^300 mg allopurinol dose POST session for Rutherford et al*.* [32], once daily for the remaining studies [29, 35]; ^¥^≈100 mg/day allopurinol dose [22]; ^$^200 mg/day allopurinol dose [29, 34, 36]; ^ǿ^350 mg/day allopurinol dose [13]. Dashed lines indicate the target urate concentration of 0.36 mmol/L in absence of tophi

A total of 997 serum urate concentrations were obtained, 662 concentrations from 99 patients receiving haemodialysis and 335 concentrations from 25 patients receiving peritoneal dialysis. Over a haemodialysis session, serum urate concentrations decreased by 56–71% in six patients (Fig. 2a, b). The dialytic clearance of urate with haemodialysis ranged from 80 to 165 mL/min at blood flow rates < 400 mL/min (*n* = 19) [24, 37]. Urate dialysate concentrations ranged from 0.12 to 0.56 mmol/L in 21 patients [25, 37]. Over time (1–230 days), serum urate concentrations in patients (*n* = 85) on haemodialysis receiving allopurinol reduced by 14–41% (Fig. 2c, d). Only one study reported the association between serum urate reduction and haemodialysis characteristics where a reduction in serum urate by ≥ 80% was associated with a blood flow rate > 400 mL/min in 69/96 patients and Kt/V > 1.3 in 35/96 patients [24].

In patients receiving peritoneal dialysis, serum urate concentrations decreased by 13.5% over 660 days [25]. The mean CAPD urate dialytic clearance ranged from 2.7–4 mL/min (*n* = 25) [23, 25] which was similar to that for continuous cyclic peritoneal dialysis (CCPD), 3.7 mL/min (*n* = 20) [25]. By contrast, the urate dialytic clearance with night intermittent peritoneal dialysis (NIPD) was lower at 1.9 mL/min (*n* = 20) [25]. The mean renal clearance of urate in these 25 patients ranged from 0.7 to 1.4 mL/min [23, 25]. As the renal clearance decreased (from 1.4 to 0.7 mL/min), the peritoneal dialytic clearance increased (from 2.7 to 4.19 mL/min) to a similar extent [23, 25].

### Target serum urate achievement

An average allopurinol dose of 121 ± 62 mg/day (haemodialysis) and 110 ± 72 mg/day (peritoneal dialysis) achieved target serum urate concentrations (< 0.36 mmol/L) in 61% (20/33) and 47% (13/28) of haemodialysis and peritoneal dialysis patients, respectively. These patients had an average serum urate concentration ranging from 0.38 to 0.39 mmol/L before commencing dialysis, and only three patients had tophi [22]. A slightly higher average allopurinol dose of 239 mg/day (dose range 200–350 mg/day) achieved target serum urate concentrations in a gout patient (case report) with similar serum urate concentrations (0.3–0.39 mmol/L) but extensive tophi prior to commencing haemodialysis [13]. In another four patients on haemodialysis [29, 34–36], ≤ 30% of the urate concentrations measured were within target despite the average daily allopurinol doses ranging from 200 to 409 mg/day (Table 3). These patients had higher serum urate concentrations (0.5–0.8 mmol/L) (Table 5) prior to starting dialysis and all had tophaceous gout.

**Table 5:** Allopurinol effectiveness in terms of urate lowering in patients on allopurinol receiving dialysis

Study (n = 16)	Urate concentration				
	Blood sampling time	PRE dialysis (mmol/L)	POST dialysis (mmol/L)	Period of observation	Decrease in concentration
Haemodialysis studies					
Alkilany et al*.,* 2022 [64]	PRE, POST	0.502 (0.393- 0.607)^a^	0.237 (0.175–0.299)^a^	1–4 years	0.265 (0.143 to 0.386)^a^ mmol/L (52%)
Arenas et al*.,* 2021 [24]	PRE, POST the 2nd HD of the week	0.309 ± 0.062^b^	0.054 ± 0.024^b^	Dialysis session	80.2% (78.4–82.0)^a^
Elion et al*.,* 1980 [27]	At 0, 2, 6 h of dialysis	0.397 ± 0.14^c^	0.204 ± 0.08^c^	Dialysis session	53 ± 5%^c^
Hayes et al*.,* 1965 [37]	PRE, POST	0.268 ± 0.06^d^	0.077 ± 0.03^d^	Dialysis session	0.19 ± 0.071 mmol/L (71%)
Hsu et al*.*, 2004 [34]^e^	NR	0.528 ± 0.09^f^	0.437 ± 0.01^f^	3 years	17%^f^
Johnson et al*.,* 1979 [36]^e^	NR	0.53 ± 0.08^f^	0.39 ± 0.1^f^	8 years	26%^f^
Matsuda et al*.*, 1993 [35]^e^	NR	0.607 ± 0.08^f^	0.464 ± 0.03^f^	2 years	23.5%^f^
Reiter et al*.,* 1998 [29]^e^	PRE, POST	0.482^g^	0.173^g^	Dialysis session	64%
Richard O. Day et al*.,* 2012 [13]^e^	PRE, POST	0.39^h^, 0.3^i^, 0.3^i^	0.17^h^	Dialysis session	0.22^h^ mmol/L (56%)
Rohn et al*.,* 2020 [26]	Baseline^j^, annually	NR	NR	NR	NR
Rutherford et al*.,* 2021 [32]	Baseline, 6 weeks, 6, 9, 12 months (PRE session)	0.365 ± 0.086	0.322 ± 0.169	12 months	0.043 ± 0.083 mmol/L (12%)
Shelmadine et al*.,* 2009 [28]	Baseline, after 3 months	0.577 (0.535- 0.767)^k^	0.345 (0.226- 0.654)^k^	3 months	0.21 ± 0.143 mmol/L (40%)
Peritoneal dialysis studies					
Diez et al*.*, 2021 [25]	2 years before, right before the 1^st^ PD session, after 1 month of PD	0.42 (0.34–0.46), 0.37 (0.3–0.42)^l^	0.32 (0.27–0.35)^l^	1 month	Median decrease by 13.5%
Wilson et al*.*, 2024 [23]	Just before, then 2, 4, 6, 8, 10, 24 h after the dose	0.24–0.39^m^	NR	NR	NR
Haemodialysis and peritoneal dialysis studies					
Ohno et al*.,* 2005 [38]	PRE^n^ (HD) or any time point sampling (CAPD)	0.5 ± 0.1^o^	NR	NR	NR
Yeo et al*.,* 2019 [22]	PRE (HD) or any time point sampling (PD)	HD: 0.38 ± 0.08, PD: 0.39 ± 0.09	0.224 ± 0.053^p^, 0.334 ± 0.079^p^	24, then 48 h after HD	Mean decrease by 41%^p^ and 12%^p^

Data represented as mean ± SD unless specified otherwise

*HD* haemodialysis, *CAPD* continuous ambulatory peritoneal dialysis, *PD* peritoneal dialysis

^a^Mean (95% confidence interval)

^b^Values for the total cohort (*n* = 96) including 17% allopurinol users

^c^Calculated mean ± SD concentrations and percentage of urate concentration decrease for 3 patients

^d^For patient T.H

^e^Case report

^f^Mean ± SD urate concentrations and percentage of concentration decrease calculated using concentrations digitized from depicted only figures

^g^Values obtained in the last year of his life (deteriorated condition)

^h^On 250 mg allopurinol dose

^i^On 350 mg allopurinol dose

^j^Defined as 3 months after dialysis initiation

^k^Median (range)

^l^Median (interquartile range), PRE values are for the 13 hyperuricemic patients including the 3 gout patients on allopurinol, while dialysate and POST values are for the total cohort (20 patients)

^m^Baseline serum urate concentration at study initiation (before dwell 1)

^n^At the beginning of the week

^o^For the total cohort including 47% (158/334) allopurinol users

^p^Calculated for haemodialysis using the reduction percentage at 24 h then 48 h based on a previous study [33]

Preliminary data in a small number of patients receiving peritoneal dialysis showed higher urate target attainment with CCPD (11/11 patients) compared to CAPD (3/6 patients) and NIPD (1/3 patients). The same study showed that the target urate achievement was significantly higher with peritoneal dialytic clearance of 3.2 than 2 mL/min/1.73 m^2^ [25].

### Frequency of gout flares

The frequency of gout flares decreased from 2 to 0.1 per year in 79 gout patients among a total cohort receiving either haemodialysis (84%) or CAPD (16%) within 2 years of commencing dialysis [38]. A case report for a patient on haemodialysis showed a similar trend, with the frequency of gout flares decreasing from 10 to 0.7 per year within 6 years of commencing dialysis [36]. Two patients were reported to have a transient increase in the frequency of gout flares in the first year of haemodialysis [35, 36].

### Adverse drug reactions

None of the included patients reported serious adverse events to allopurinol with doses up to 400 mg/day being used [13, 37].

## Discussion

In this scoping review, we observed that allopurinol dose requirements differed by the dialysis modality. Urate and oxypurinol were removed at a similar extent in each dialysis modality with higher clearances by haemodialysis than peritoneal dialysis. Attainment of target serum urate concentrations was associated with lower urate concentrations prior to beginning dialysis, higher allopurinol doses, and the absence of tophi. During a haemodialysis session, 39–57% of oxypurinol is cleared, a finding that supports the administration of allopurinol after haemodialysis sessions with up-titration of doses according to the treat-to-target approach. Additional research is required to fully understand the impact of dialyser type and dialysis conditions, such as blood and dialysate flow rates, on the pharmacokinetics of oxypurinol and the attainment of the urate target. As for peritoneal dialysis, more evidence is needed to inform the optimal dosing of allopurinol for gout patients receiving this dialysis modality, since it may differ in those receiving intermittent exchanges with ‘dry’ periods (i.e., NIPD).

Haemodialysis was more effective in removing oxypurinol, with a dialytic clearance 25–44 times greater, than peritoneal dialysis. This reflects the higher efficiency of haemodialysis compared to peritoneal dialysis [24, 25]. Given that these dialysers clear up to 57% of the 'dose' of oxypurinol during a single haemodialysis session, allopurinol should ideally be administered after dialysis to ensure adequate exposure during the interdialytic period; a practice consistent with current guidelines (Table S1, Online resource 1) and product information leaflets [39–41]. Alternatively, if administered before the dialysis session, the dose of allopurinol would need to be doubled to account for the oxypurinol clearance during the dialysis session. Interestingly, the haemodialytic clearance of oxypurinol has approximately doubled in more recent studies compared to older studies, despite similar blood flow rates. This likely reflects the advancements in dialyser technology over time. For example, the study by Doogue et al. [33] used the polysulfone dialyzer which has a higher efficiency compared to the Kiil and Gambro dialyzers used by Hande et al. [8] and Hayes et al. [37]. The use of polysulfone dialysers is currently standard practice [42, 43]. Whilst there are some data on current haemodialysis dialysers (e.g. polysulfone dialyser), as dialyser technology evolves and newer dialysers such as the Fresenius FX class^®^ or Filtryzer^®^ [42] are used more commonly, studies on how efficiently these newer dialysers clear urate and oxypurinol compared to older dialysers will be required to assess whether adjustments to allopurinol dosing are required. The influence of other factors related to dialysers, such as dialysis flux and blood and dialysate flow rates, may also impact hemodialytic clearance [44]. More research is required to understand the effect of these factors on allopurinol dose requirements. Regarding peritoneal dialysis, this modality remains relatively underexplored; specifically, the impact of dwell time and volume of fluid exchange on oxypurinol pharmacokinetics and the urate-lowering response to allopurinol remain unknown. Furthermore, the pharmacokinetics of oxypurinol in patients receiving automated peritoneal dialysis or intermittent dwells with dry periods is unknown and warrants further investigation to inform optimal dosing.

Similar to oxypurinol, urate clearance was much higher with haemodialysis compared to peritoneal dialysis. Again, this difference is attributed to the higher efficiency of haemodialysis relative to peritoneal dialysis. Oxypurinol and urate have similar structures and small molecule size [45, 46]. This may explain why they are handled similarly by dialysis modalities [47]. Although serum urate concentrations decreased during a haemodialysis session, they increased during the interdialytic period, returning to the levels prior to the dialysis session. Therefore, PRE dialysis serum urate concentration monitoring to determine target concentration attainment should be performed regularly. Lastly, the impact of CRRT on oxypurinol pharmacokinetics or effectiveness in people with gout remains unknown. Although it is acknowledged that optimisation of allopurinol dosing in critically ill patients receiving CRRT may be less of a priority than other lifesaving measures, understanding the impact of this dialysis modality on allopurinol dose requirements is necessary.

Although data were limited, according to the largest study to date, that included 33 patients on haemodialysis and 28 on peritoneal dialysis, the achievement of target serum urate concentrations was associated with serum urate concentration before starting dialysis, allopurinol dose, and the presence of tophi. These factors have previously been associated with the achievement of target urate in patients with gout who receive allopurinol but do not receive dialysis [48–53]. In one of the largest studies conducted in 17,402 patients with gout, the probability of achieving target serum urate concentrations was 40–65% lower in patients with a baseline serum urate concentration greater than 0.50 mmol/L, and 3–17% higher with maintenance allopurinol doses greater than 100 mg/day [50]. Consistent with this, less than 30% of urate concentrations for four patients [29, 34–36] with baseline urate ≥ 0.50 mmol/L, tophaceous gout, were within the target despite receiving allopurinol doses > 100 mg/day. The allopurinol dose required to reach the target concentrations of urate was higher in patients receiving haemodialysis compared to peritoneal dialysis, which is expected due to greater dialytic clearance of oxypurinol in haemodialysis. Additionally, data from two patients receiving haemodialysis demonstrate that target urate concentrations can be achieved if allopurinol doses are escalated [13, 36]. This suggests that the treat-to-target approach should also be applied to patients with gout who receive dialysis [54].

There are insufficient data to determine whether dialysis conditions (e.g. blood flow rate or dwell time) influence the attainment of target urate concentrations in gout patients receiving dialysis. For example, higher blood flow rates are expected to result in greater reductions in serum urate (and oxypurinol) concentrations, as demonstrated by Arenas et al*.* [24]. However, the effect of dialysis conditions (e.g. increasing dialysis intensity) on the achievement of target urate, especially in those with tophaceous gout, requires further research.

Despite its relative inefficiency in urate removal, the continuous nature of peritoneal dialysis appears to overcome this deficiency, at least for patients with non severe tophaceous gout. Preliminary data indicate that the modality of peritoneal dialysis may also affect the target urate attainment for patients with gout [25]. Additional studies to investigate the impact of peritoneal dialysis modality on the achievement of target urate concentrations are therefore required. The ability of haemodialysis to remove urate more effectively suggests that it is the most suitable modality to achieve target urate concentrations in patients with severe tophaceous gout.

The effect of allopurinol and dialysis on gout flares has been examined in one study of 413 haemodialysis and 80 CAPD patients, 79 of whom had gout [38]. Over a period of 2 years, the frequency of gout flares was significantly reduced. A case report of two patients, demonstrated a transient increase in the frequency of gout flares after commencing haemodialysis [35, 36]. This likely reflects the sudden lowering of serum urate concentrations. Fluctuations in serum urate concentrations are known to precipitate gout flares [55–57]. Thus, prophylaxis with NSAIDs or colchicine is recommended when starting dialysis.

This is the first scoping review focusing on allopurinol dosing and effectiveness in gout patients on any dialysis modality. Previous reviews included dialysis patients as a subset of the population [58, 59] but did not evaluate this patient population in detail. This review has some limitations. Firstly, details on the dialysis modality, administration of allopurinol and sampling of oxypurinol and urate in relation to dialysis were often not reported. This information is required to enable the assessment of clearance rates and target serum urate achievement. Future studies should also report on the technical aspects of dialysis (dialyzer types, blood flow rates, and hours and frequency of dialysis treatments, etc.) to facilitate the applicability of the findings and enable clinicians to create individualized dose strategies for patients receiving dialysis. The change in serum urate concentrations over a peritoneal dialysis dwell could not be calculated due to lack of information. Similarly, there was not enough information to support timing of allopurinol administration in relation to the dwells in patients on peritoneal dialysis. Theoretically, unless peritoneal dialysis is administered intermittently (with ‘dry’ spells), allopurinol administration before or after a dwell is not expected to change the pharmacokinetics or affect dosing. As for haemodialysis, the POST dialysis session concentrations might have been confounded by concentration rebound that usually occurs within 30–90 min. after the dialysis session [60]. The reported serum target urate achievement was based on the largest available study by Yeo et al. [22]. Due to the cross-sectional nature of the study, the time on dialysis differed between patients. However, the mean time on dialysis (1399 days; approximately 4 years) was sufficient to assess the impact of dialysis and allopurinol on achievement of target urate levels in accordance with guidelines, which recommend assessment over a 12-month period [61]. Future prospective studies with standardized reporting of outcomes are needed to confirm these findings.

Secondly, due to the small sample sizes, to the heterogeneity of the dialysis modalities and conditions used, and to the development of dialysis technology over time, the comparison of findings between studies should be carefully considered. As some of the data required digitisation, this could have introduced errors. Lastly, due to the paucity of data, we included case reports and case series which carry a potential risk of bias. However, previous reviews in dialysis patients have also included case reports [62, 63].

## Conclusions

Dosing of allopurinol in patients with gout who receive haemodialysis or peritoneal dialysis is challenging because both urate and oxypurinol are cleared by dialysis. In patients receiving haemodialysis, allopurinol should be dosed after the session, as almost half of the dose is cleared during dialysis. Higher allopurinol dose, lower serum urate concentrations before starting dialysis, and the absence of tophi are associated with greater probability of achieving serum urate targets. Consequently, escalation of allopurinol doses is recommended until the serum urate target is achieved. Due to large fluctuations in serum urate concentrations, prophylaxis is suggested to prevent gout flares when dialysis is started. These approaches are consistent with practice in patients with gout who do not receive dialysis. Additional research is required to determine the dialysis conditions that contribute to variability in response to allopurinol to inform dose individualisation. Quantification of these factors, using a modelling and simulation approach, can assist in identifying optimal allopurinol dosing strategies in different dialysis populations.

## Supplementary Information

Below is the link to the electronic supplementary material.

Supplementary file1 (DOCX 51 KB)

Supplementary file2 (DOCX 80 KB)

## Declarations

### Data availability

Data sharing is not applicable to this article as no datasets were generated or analysed during the current review.
