# Supplementary material for: Dosing practices, pharmacokinetics, and effectiveness of allopurinol in gout patients receiving dialysis: a scoping review
Source: J Nephrol. 2025 Mar 25;38(3):859–75. doi: 10.1007/s40620-025-02269-7 (PMC12166007; doi:10.1007/s40620-025-02269-7)
Supplement: Supplementary file 1 — Supplementary file1 (DOCX 51 KB) [file 40620_2025_2269_MOESM1_ESM.docx]

**Article title:**  Dosing practices, pharmacokinetics, and effectiveness of allopurinol in gout patients receiving dialysis: a scoping review

**Journal name:** Journal of Nephrology

**Author names:** Noha A. Kamel, Michael A. Stokes, Daniel F.B. Wright, Kamal Sud, Surjit Tarafdar, Ronald L. Castelino, Sophie L. Stocker

**Corresponding author:** Dr. Sophie Stocker, School of Pharmacy, Faculty of Medicine & Health, Univ. of Sydney, Australia, [sophie.stocker@sydney.edu.au](mailto:sophie.stocker@sydney.edu.au)

# **Online resource 1: Details of studies reporting allopurinol effectiveness outcomes and summary of the only population pharmacokinetic model in literature for oxypurinol in haemodialysis**

## **Table S1. Summary for guideline recommendations for allopurinol dosing in stages 4 and 5 of chronic kidney disease and dialysis**

|  | **UpToDate [1]** | | **Renal drug handbook [2]^a^** | | **British society of rheumatology [3]** | | **Australian medicine handbook [4]** | | **European alliance of associations for rheumatology [5]** | | **American college of rheumatology [6]** | |
| --- | --- | --- | --- | --- | --- | --- | --- | --- | --- | --- | --- | --- |
|  | Initial dose | Titration increment | Initial dose | Maintenance dose | Initial dose | Titration | Initial dose | Titration | Initial dose | Maintenance dose^b^ | Initial dose | Titration |
| **Stage 4 (eGFR 15-29 ml/min/1.73 m^2^)** | 50 mg on alternative days | ≤ 100 mg/day q 2-4 w or ≤ 50 mg/day q ≥ 4w (preferred) to the minimum dose achieving target urate concentrations^d^ | 100 mg/day | 100-200 mg/day according to target urate achievement | 50 mg on alternative days | 50 mg increments till achieving target urate of 0.3 mmol/l | 50 mg on alternative days^c^ | Slowly titrate the dose to achieve target urate^d^ | low^e^ | 100 mg q 2d^f^ or 100 mg/day^g^ | ≤50 mg/day | To target with the schedule for increments left to the physician and patient^d,i^ |
| **Stage 5 (eGFR < 15 ml/min/1.73 m^2^)** | 50 mg twice/w for eGFR 5-15, once/w for eGFR <5 ml/min/1.73 m^2^ |  | 100 mg/day or 100 mg on alternative days | 100 mg/day or 100 mg on alternative days according to target urate achievement | 50 mg twice/w for eGFR 5-15, once/w for eGFR <5 ml/min/1.73 m^2^ |  | 50 mg twice/w^c^ |  | low^e^ | 100 mg q 2d^f^ or 100 mg q 3d^h^ |  |  |
| **Haemodialysis** | 100 mg thrice/w post-dialysis | ≤ 50 mg/day q 2-5 w to the minimum dose achieving target urate concentrations ^d^ | As stage 5 | As stage 5 or 300-400 mg post session on dialysis days only | No recommendations | | No recommendations | | No recommendations | | No recommendations | |
| **Peritoneal dialysis** | 50 mg/day |  | As stage 5 (CAPD, APD) | As stage 5 (CAPD, APD) | No recommendations | | No recommendations | | No recommendations | | No recommendations | |

**eGFR:** estimated glomerular filtration rate; **mg/day q 2-4 w:** milligrams per day every 2-4 weeks; **CAPD:** continuous ambulatory peritoneal dialysis; **APD:** automated peritoneal dialysis; ^a^: defines stage 4 as GFR 10-20 ml/min and stage 5 as GFR <10 ml/min ^b^: if the target urate cannot be achieved with this maintenance dose, the patient should be switched to febuxostat or benzbromarone unless the e GFR is <30 ml/min; ^c^: calculated as 1.5*e GFR (ml/min/1.73 m^2^); ^d^: titration can be delayed for the first 1-2 months until the peak risk period for AHS passes (expert opinion), maintenance doses > 300 mg can be used with proper patient education and monitoring for toxicity; ^e^: exact dose not mentioned; ^f^: for creatinine clearance of 10 ml/min; ^g^: for creatinine clearance of 20 ml/min;. ^h^: for creatinine clearance of 0 ml/min; ^i^: the titration schedule can be individualized according to the patient’s preference and comorbidities as long as it occurs over a suitable time frame (weeks to months).

## **Table S2. Summary of Wright *et al.*’s population pharmacokinetic model of oxypurinol in gout patients on haemodialysis [7]**

| **Structural model** | | | | | | | | | | | | | | | | | | | | | | | | | | | |
| --- | --- | --- | --- | --- | --- | --- | --- | --- | --- | --- | --- | --- | --- | --- | --- | --- | --- | --- | --- | --- | --- | --- | --- | --- | --- | --- | --- |
| **CMT** | **Absorption** | **Elimination** | | | | | **Variance model** | | | | | | | | **Dialytic clearance** | | | | | **Model selection criteria** | | | | | **Covariates** | | |
| 1 CMT | 1^st^ order | 1^st^ order | | | | | Combined (additive & proportional) residual variance model | | | | | | | | Separate parameter using a flag to turn dialysis on and off | | | | | Guided by OFV dec, GOF plots, VPC, parameter precision, biological plausibility | | | | | FFM, RF for $\frac{Cl}{Foxy}$and TBW for $\frac{V}{Foxy}$ | | |
| **Variability** | | | | | | | | | | | | | | | | | | | | | | | | | | | |
| **Distribution** | **BSV estimate (RSE %)** | | | | | | | | | | | | **Residual error estimate (RSE %)** | | | | | | | | | **Shrinkage** | | | | | **covariance** |
|  | **BSV (CLoxy)** | | | | **BSV (Voxy)** | | | | **BSV (Ka)** | | | | **Proportional** | | | | | | | **Additive** | | **η shrinkage** | | | **ℇ shrinkage** | |  |
|  |  |  |  |  |  |  |  |  |  |  |  |  | **Oxypurinol dialysis^a^** | | | | | **Allopurinol starter^b^** | | **fixation** | | **CL_oxy_** | **V_oxy_** | **ka** |  |  |  |
| Log-normal | ωCLoxy (CV%): 34.5 (45.0) | | | | ωVoxy (CV%): 20.9 (48.8) | | | | ωKa (CV%): 75.9 (35.5) | | | | σ_prop_ (CV%): 10.1 (14.2) | | | | | σ_prop_ (CV%): 21.2 (12.3) | | σ_add_ was fixed at 0.001 | | 4.8 % | 8.4 % | 5.9 % | 10.83 % | | ηCLoxy, ηVoxy: 0.029 |
| **Simulations** | | | | | | | | | | | | | | | | | | | | | | | | | | | |
| **Dialysis** | | | | | | | | | | | | | | | | | **Without dialysis** | | | | | | | | | | |
| **Allopurinol doses** | **Duration** | | | **Regimen** | | **Dosing times** | | | | **TBW** | | **GFR** | | **Diuretic therapy?** | | | **Allopurinol doses** | | **Duration** | | | **TBW** | **GFR** | | | **Diuretic therapy?** | |
| 100 mg/week, 100 mg/day and 100 mg q 2d | Simulations conducted for 28 d | | | 3 times/w | | PRE^c^, POST^d^ | | | | 70 kg | | 2 mL/min | | No | | | 100 mg/day and usual doses of 200, 300, 400 mg/day | | 28 d | | | 70 kg | For 100 mg/day dose: CrCL (mL/min) 2, 10, 20, 40, 60, 80, 100 mL/min | | | No | |
| **Model evaluation methods** | | | **Model parameters & their estimates (RSE %)** | | | | | | | | | | | | | | | | | | | | | | | | |
| Through VPC, individual fit plots, and NP-BS | | | **Inter-dialytic clearance** | | | | | **Dialytic clearance** | | | **Volume** | | | | | **Absorption rate constant** | | | | | **Renal function** | | | | | | |
|  |  |  | θCL (L/h/70 kg FFM/CLcr 6 L/h): 1.20 (7.5) | | | | | θCLHD (L/h): 8.23 (7.0) | | | θV (L/70 kg TBW): 48.7 (5.7) | | | | | Ka (h^−1^): 0.941 | | | | | θ _RFexp_: 0.54 (9.0) | | | | | | |
| **Model-predicted PK parameters^e^** | | | | | **Final model equations** | | | | | | | | | | | | | | | | | | | | | | |
| **AUC_7d_** | **Cpss_min_** | | | |  |  |  |  |  |  |  |  |  |  |  |  |  |  |  |  |  |  |  |  |  |  |  |
| 279 umol/L h | 13 umol/L | | | | $\frac{Cl}{Foxy}\left( \frac{L}{h} \right)=\left( \theta CL \times\left( \frac{CLcr (\frac{L}{h}}{6 \left( \frac{L}{h} \right)} \right)^{\theta RFexp} \right) \times{(\frac{FFM}{70 kg})}^{0.75}$  ${CLHD}_{oxy}\left( \frac{L}{h} \right)= \theta_{CLHD}$  $\frac{V}{Foxy}\left( L \right)= \theta_{v} \times(\frac{TBW}{70 kg})$ | | | | | | | | | | | | | | | | | | | | | | |

**CMT:** compartment; **OFV dec:** objective function value decrease; **GOF plots:** goodness of fit plots; **VPC:** visual predictive check; **FFM:** fat free mass; **RF:** renal function; **Cl/Foxy:** apparent oxypurinol clearance; **TBW:** total body weight; **V⁄Foxy:** apparent oxypurinol volume of distribution; **BSV:** between subject variability; **RSE:** relative standard error; **CL:** clearance; **V:** volume of distribution; **Ka:** absorption rate constant; **CV%:** coefficient of variation; **GFR:** glomerular filtration rate; **CrCL:** creatinine clearance; **NP-BS:** non-parametric bootstrap; **CLHD:** dialytic clearance during haemodialysis; **AUC_7d_:** area under the plasma concentration time curve for 1 week; **Cp_ssmin_:** steady-state trough concentration.^a^: study on 6 gout haemodialysis patients on allopurinol whose data were used to build the model; ^b^: study on 19 gout, non-dialytic patients on allopurinol whose data were used to estimate the inter-dialytic clearance; ^c^: Pre-dialysis, just prior to dialysis start, ^d^:post-dialysis, just after dialysis end; ^e^ For 100 mg/day allopurinol given post-dialysis.

## **References**

1. UpToDate. (2023) *Allopurinol: Drug information*. <https://www.uptodate.com/contents/allopurinol-drug-information?source=mostViewed_widget>. Accessed 28th Nov 2023.

2. Ashley C and Dunleavey A, *The renal drug handbook: the ultimate prescribing guide for renal practitioners*. 5th edition. ed. 2019, Boca Raton, FL: CRC Press.

3. Hui M, Carr A, Cameron S, Davenport G, Doherty M, Forrester H, Jenkins W, et al (2017) *The British Society for Rheumatology Guideline for the Management of Gout.* Rheumatology (Oxford). **56**:e1-e20. <https://doi.org/10.1093/rheumatology/kex156>

4. AMH. (2023) *Allopurinol*. <https://amhonline-amh-net-au.ezproxy.library.sydney.edu.au/chapters/rheumatological-drugs/drugs-gout/xanthine-oxidase-inhibitors/allopurinol>. Accessed 28th Nov 2023.

5. Richette P, Doherty M, Pascual E, Barskova V, Becce F, Castañeda-Sanabria J, Coyfish M, et al (2017) *2016 updated EULAR evidence-based recommendations for the management of gout.* Ann Rheum Dis. **76**:29-42. <https://doi.org/10.1136/annrheumdis-2016-209707>

6. FitzGerald JD, Dalbeth N, Mikuls T, Brignardello-Petersen R, Guyatt G, Abeles AM, Gelber AC, et al (2020) *2020 American College of Rheumatology Guideline for the Management of Gout.* Arthritis Care Res (Hoboken). **72**:744-760. <https://doi.org/10.1002/acr.24180>

7. Wright D, Doogue M, Barclay M, Chapman P, Cross N, Irvine J, and Stamp L (2017) *A population pharmacokinetic model to predict oxypurinol exposure in patients on haemodialysis.* Eur J Clin Pharmacol. . **73**:71-78. <https://doi.org/10.1007/s00228-016-2133-y>
