# Supplementary material for: Dosing practices, pharmacokinetics, and effectiveness of allopurinol in gout patients receiving dialysis: a scoping review
Source: J Nephrol. 2025 Mar 25;38(3):859–75. doi: 10.1007/s40620-025-02269-7 (PMC12166007; doi:10.1007/s40620-025-02269-7)
Supplement: Supplementary file 2 — Supplementary file2 (DOCX 80 KB) [file 40620_2025_2269_MOESM2_ESM.docx]

**Article title:** Dosing practices, pharmacokinetics, and effectiveness of allopurinol in gout patients receiving dialysis: a scoping review

**Journal name:** Journal of Nephrology

**Author names:** Noha A. Kamel, Michael A. Stokes, Daniel F.B. Wright, Kamal Sud, Surjit Tarafdar, Ronald L. Castelino, Sophie L. Stocker

**Corresponding author:** Dr. Sophie Stocker, School of Pharmacy, Faculty of Medicine & Health, Univ. of Sydney, Australia, [sophie.stocker@sydney.edu.au](mailto:sophie.stocker@sydney.edu.au)

# **Online resource 2: Detailed search strategy for all databases and clinical trial registries**

### **Embase**

| **#** | **Query** |
| --- | --- |
| 1 | Allopurinol*.tw,kf. |
| 2 | antigout agent/ or exp allopurinol/ |
| 3 | (suppressant* and gout*).tw,kf. |
| 4 | (Agent* and Antigout*).tw,kf. |
| 5 | (Antihyperuricemic* or Anti-hyperuricemic*).tw,kf. |
| 6 | xanthine oxidase inhibitor/ or exp allopurinol/ |
| 7 | (urate* lower* therap* or uric acid* lower* therap*).tw,kf. |
| 8 | ((therap* or treatment*) adj3 (uric acid* or urate*)).tw,kf. |
| 9 | Xanthine oxidase inhibitor*.tw,kf. |
| 10 | ((inhibit* or deactivat* or inactivat*) adj3 xanthine oxidase*).tw,kf. |
| 11 | Gout* Suppressant*.tw,kf. |
| 12 | Antigout* Agent*.tw,kf. |
| 13 | Zyloric*.tw,kf. |
| 14 | Uricto*.tw,kf. |
| 15 | Allosig.tw,kf. |
| 16 | Progout*.tw,kf. |
| 17 | Zyloprim*.tw,kf. |
| 18 | (1 or 2 or 3 or 4 or 5 or 6 or 7 or 8 or 9 or 10 or 11 or 12 or 13 or 14 or 15 or 16 or 17) not rasburicase*.tw,kf. |
| 19 | dialysis/ or exp extended daily dialysis/ or exp hemodiafiltration/ or exp hemodialysis/ or exp peritoneal dialysis/ |
| 20 | dialysis fluid/ or exp peritoneal dialysis fluid/ |
| 21 | extracorporeal therapy device/ or exp "dialysis equipment and supplies"/ or exp hemofiltration system/ |
| 22 | renal replacement therapy/ or exp continuous renal replacement therapy/ or exp extended daily dialysis/ or exp hemodiafiltration/ or exp hemodialysis/ or exp hemofiltration/ or exp intermittent renal replacement therapy/ or exp peritoneal dialysis/ |
| 23 | "filters and membranes"/ or exp dialysis membrane/ |
| 24 | pump/ or exp dialysis pump/ |
| 25 | peritoneal catheter/ or exp peritoneal dialysis catheter/ |
| 26 | exp hemodialysis patient/ |
| 27 | he?modialys*.tw,kf. |
| 28 | Dialys*.tw,kf. |
| 29 | (dialys* adj3 (kidney* or renal* or peritoneal* or fluid* or patient* or end stage renal disease* or end-stage renal disease*)).tw,kf. |
| 30 | renal replacement* therap*.tw,kf. |
| 31 | (19 or 20 or 21 or 22 or 23 or 24 or 25 or 26 or 27 or 28 or 29 or 30) not transplant*.tw,kf. not leuk*emia*.tw,kf. not lymphoma*.tw,kf. not tumor* lys*.tw,kf. |
| 32 | exp pharmacokinetics/ |
| 33 | exp pharmacokinetic parameters/ |
| 34 | blood level/ or drug blood level/ |
| 35 | exp pharmacokinetic modeling software/ |
| 36 | "concentration (parameter)"/ or exp concentration ratio/ or exp drug concentration/ or exp inhibitory concentration/ or exp lethal concentration/ or exp lowest-observed-adverse-effect concentration/ or exp lowest-observed-effect concentration/ or exp maximum concentration/ or exp minimum concentration/ or exp no-observed-adverse-effect concentration/ or exp no-observed-effect concentration/ or exp toxic concentration/ or exp trough concentration/ |
| 37 | exp oxipurinol/ |
| 38 | dialysate level/ |
| 39 | pharmacokinetic*.tw,kf. |
| 40 | ((pharmacokinetic* or non-linear mixed effect* or nonlinear mixed effect* or pharmacometric*) adj3 (Model* or simulat* or parameter*)).tw,kf. |
| 41 | dialy* clearance*.tw,kf. |
| 42 | (Drug* adj3 (level* or concentration*)).tw,kf. |
| 43 | Population* pharmacokinetic*.tw,kf. |
| 44 | (Population* adj3 pharmacokinetic*).tw,kf. |
| 45 | NLME*.tw,kf. |
| 46 | NONMEM*.tw,kf. |
| 47 | Ox#purinol*.tw,kf. |
| 48 | Personali#ed dos*.tw,kf. |
| 49 | Individuali#ed dos*.tw,kf. |
| 50 | Precis* dos*.tw,kf. |
| 51 | (Dos* adj3 (Personali#ed or Individuali#ed or Precis*)).tw,kf. |
| 52 | 32 or 33 or 34 or 35 or 36 or 37 or 38 or 39 or 40 or 41 or 42 or 43 or 44 or 45 or 46 or 47 or 48 or 49 or 50 or 51 |
| 53 | exp pharmacodynamics/ |
| 54 | exp pharmacodynamic parameters/ |
| 55 | uric acid derivative/ or exp urate/ or exp uric acid/ |
| 56 | exp uric acid blood level/ |
| 57 | adverse event/ or exp adverse drug reaction/ |
| 58 | disease exacerbation/ |
| 59 | pharmacodynamic*.tw,kf. |
| 60 | dos* respon*.tw,kf. |
| 61 | ((Dos* or drug*) adj3 (respon* or eff#c*)).tw,kf. |
| 62 | Drug* eff#c*.tw,kf. |
| 63 | Population* pharmacodynamic*.tw,kf. |
| 64 | ((Population* or model*) adj3 pharmacodynamic*).tw,kf. |
| 65 | pharmacodynamic* model*.tw,kf. |
| 66 | ((uric acid* or urate*) adj3 (level* or concentration* or target*)).tw,kf. |
| 67 | Uric acid*.tw,kf. |
| 68 | Urate*.tw,kf. |
| 69 | "PK*/PD*".tw,kf. |
| 70 | "pharmacokinetic*/pharmacodynamic*".tw,kf. |
| 71 | ((Gout* flare* or gout* attack*) adj3 (frequen* or number* or mean* or median*)).tw,kf. |
| 72 | ((metabolite* or drug*) adj3 expos*).tw,kf. |
| 73 | drug exposure/ or exp drug reexposure/ |
| 74 | ((adverse event* or adverse rection* or side effect*) adj3 (number* or frequen* or mean* or median*)).tw,kf. |
| 75 | 53 or 54 or 55 or 56 or 57 or 58 or 59 or 60 or 61 or 62 or 63 or 64 or 65 or 66 or 67 or 68 or 69 or 70 or 71 or 72 or 73 or 74 |
| 76 | 52 or 75 |
| 77 | 18 and 31 and 76 |
| 78 | limit 77 to human |
| 79 | limit 78 to dc=20231019-20240501 |

### **Scopus**

| **Concept and Query** |
| --- |
| **Allopurinol concept** |
| ( TITLE-ABS-KEY ( allopurinol* ) OR TITLE-ABS-KEY ( suppressant* AND gout* ) OR TITLE-ABS-KEY ( agent* AND antigout* ) OR TITLE-ABS-KEY ( antihyperuricemic* OR "Anti-hyperuricemic*" ) OR TITLE-ABS-KEY ( "urate* lower* therap*" OR "uric acid* lower* therap*" ) OR TITLE-ABS-KEY ( ( therap* OR treatment* ) W/2 ( "uric acid*" OR urate* ) ) OR TITLE-ABS-KEY ( "Xanthine oxidase inhibitor*" ) OR TITLE-ABS-KEY ( ( inhibit* OR deactivat* OR inactivat* ) W/2 ( "xanthine oxidase*" ) ) OR TITLE-ABS-KEY ( "Gout* Suppressant*" ) OR TITLE-ABS-KEY ( "Antigout* Agent*" ) OR TITLE-ABS-KEY ( zyloprim* ) OR TITLE-ABS-KEY ( lopurin* ) OR TITLE-ABS-KEY ( aloprim* ) OR TITLE-ABS-KEY ( zyloric* ) OR TITLE-ABS-KEY ( uricto* ) OR TITLE-ABS-KEY ( allosig ) OR TITLE-ABS-KEY ( progout* ) AND NOT TITLE-ABS-KEY ( rasburicase* ) ) |
| **Dialysis concept** |
| ( TITLE-ABS-KEY ( "Home Dialys*" ) OR TITLE-ABS-KEY ( ( sorption* OR detoxification* ) W/2 ( blood* OR plasma* ) ) OR TITLE-ABS-KEY ( h*mofiltration* ) OR TITLE-ABS-KEY ( h*moperfusion* ) OR TITLE-ABS-KEY ( ( dialys* OR h*modialys* ) W/2 ( equipment* OR suppl* OR device* OR machine* OR catheter* OR membrane* OR center* OR patient* OR therap* OR solution* OR extracorporeal* ) ) OR TITLE-ABS-KEY ( ultrafiltration* ) OR TITLE-ABS-KEY ( h*modialys* ) OR TITLE-ABS-KEY ( dialys* ) OR TITLE-ABS-KEY ( ( dialys* ) W/2 ( kidney* OR renal* OR peritoneal* OR fluid* OR patient* OR "end stage renal disease*" ) ) OR TITLE-ABS-KEY ( *rrt* ) OR TITLE-ABS-KEY ( "renal replacement* therap*" ) AND NOT TITLE-ABS-KEY ( transplant* ) AND NOT TITLE-ABS-KEY ( leuk*mia* ) AND NOT TITLE-ABS-KEY ( lymphoma* ) AND NOT TITLE-ABS-KEY ( "tumor* lys*" ) ) |
| **PK or PD concepts** |
| ( ( TITLE-ABS-KEY ( pharmacokinetic* ) OR TITLE-ABS-KEY ( ( drug* OR metabolite* ) W/2 ( level* OR concentration* ) ) OR TITLE-ABS-KEY ( ( medicine* OR drug* OR dos* ) W/2 ( precis* OR individuali?e* OR personali?e* OR monitor* ) ) OR TITLE-ABS-KEY ( ( pharmacokinetic* OR "non-linear mixed effect*" OR "nonlinear mixed effect*" OR pharmacometric* ) W/2 ( model* OR simulat* OR parameter* OR statistic* ) ) OR TITLE-ABS-KEY ( "*drug Monitor*" OR tdm* ) OR TITLE-ABS-KEY ( "dialy* clearance*" ) OR TITLE-ABS-KEY ( "population* pharmacokinetic*" ) OR TITLE-ABS-KEY ( ( population* ) W/2 ( pharmacokinetic* ) ) OR TITLE-ABS-KEY ( nlme* ) OR TITLE-ABS-KEY ( nonmem* OR "Nonlinear Mixed Effect* Model*" OR "Population Pharmacokinetic Model*" ) OR TITLE-ABS-KEY ( pharmacometric* ) OR TITLE-ABS-KEY ( ox?purinol* ) OR TITLE-ABS-KEY ( "Personali?ed dos*" OR "Individuali?ed dos*" OR "Precis* dos*" OR "Model-informed Precision Dos*" OR "Dos* Individuali?*" ) OR TITLE-ABS-KEY ( mipd* OR "dos* strateg*" ) ) ) OR ( ( TITLE-ABS-KEY ( pharmacodynamic* ) OR TITLE-ABS-KEY ( "dos* respon*" ) OR TITLE-ABS-KEY ( ( dos* OR drug* ) W/2 ( respon* OR eff?c* ) ) OR TITLE-ABS-KEY ( "Drug* eff?c*" ) OR TITLE-ABS-KEY ( "Treatment* Outcome*" OR "Therapeutic Index" OR "Treatment* Fail*" OR "Treatment* Terminat*" OR "Fatal Outcome*" ) OR TITLE-ABS-KEY ( "Disease Exacerbation*" ) OR TITLE-ABS-KEY ( "population* pharmacodynamic*" ) OR TITLE-ABS-KEY ( ( population* OR model* ) W/2 ( pharmacodynamic* ) ) OR TITLE-ABS-KEY ( "pharmacodynamic* model*" ) OR TITLE-ABS-KEY ( ( "uric acid*" OR urate* ) W/2 ( level* OR concentration* OR target* ) ) OR TITLE-ABS-KEY ( "uric acid*" ) OR TITLE-ABS-KEY ( urate* ) OR TITLE-ABS-KEY ( "PK*/PD*" ) OR TITLE-ABS-KEY ( "Pharmacokinetic*/pharmacodynamic*" ) OR TITLE-ABS-KEY ( ( "gout* flare*" OR "gout* attack*" ) W/2 ( frequen* OR number* OR mean* OR median* ) ) OR TITLE-ABS-KEY ( ( metabolite* OR drug* ) W/2 ( expos* ) ) OR TITLE-ABS-KEY ( ( "adverse event*" OR "adverse rection*" OR "side effect*" ) W/2 ( number* OR frequen* OR mean* OR median* ) ) ) ) |
| **Allopurinol and dialysis and PK/PD** |
| ( ( TITLE-ABS-KEY ( allopurinol* ) OR TITLE-ABS-KEY ( suppressant* AND gout* ) OR TITLE-ABS-KEY ( agent* AND antigout* ) OR TITLE-ABS-KEY ( antihyperuricemic* OR "Anti-hyperuricemic*" ) OR TITLE-ABS-KEY ( "urate* lower* therap*" OR "uric acid* lower* therap*" ) OR TITLE-ABS-KEY ( ( therap* OR treatment* ) W/2 ( "uric acid*" OR urate* ) ) OR TITLE-ABS-KEY ( "Xanthine oxidase inhibitor*" ) OR TITLE-ABS-KEY ( ( inhibit* OR deactivat* OR inactivat* ) W/2 ( "xanthine oxidase*" ) ) OR TITLE-ABS-KEY ( "Gout* Suppressant*" ) OR TITLE-ABS-KEY ( "Antigout* Agent*" ) OR TITLE-ABS-KEY ( zyloprim* ) OR TITLE-ABS-KEY ( lopurin* ) OR TITLE-ABS-KEY ( aloprim* ) OR TITLE-ABS-KEY ( zyloric* ) OR TITLE-ABS-KEY ( uricto* ) OR TITLE-ABS-KEY ( allosig ) OR TITLE-ABS-KEY ( progout* ) AND NOT TITLE-ABS-KEY ( rasburicase* ) ) ) AND ( ( TITLE-ABS-KEY ( "Home Dialys*" ) OR TITLE-ABS-KEY ( ( sorption* OR detoxification* ) W/2 ( blood* OR plasma* ) ) OR TITLE-ABS-KEY ( h*mofiltration* ) OR TITLE-ABS-KEY ( h*moperfusion* ) OR TITLE-ABS-KEY ( ( dialys* OR h*modialys* ) W/2 ( equipment* OR suppl* OR device* OR machine* OR catheter* OR membrane* OR center* OR patient* OR therap* OR solution* OR extracorporeal* ) ) OR TITLE-ABS-KEY ( ultrafiltration* ) OR TITLE-ABS-KEY ( h*modialys* ) OR TITLE-ABS-KEY ( dialys* ) OR TITLE-ABS-KEY ( ( dialys* ) W/2 ( kidney* OR renal* OR peritoneal* OR fluid* OR patient* OR "end stage renal disease*" ) ) OR TITLE-ABS-KEY ( *rrt* ) OR TITLE-ABS-KEY ( "renal replacement* therap*" ) AND NOT TITLE-ABS-KEY ( transplant* ) AND NOT TITLE-ABS-KEY ( leuk*mia* ) AND NOT TITLE-ABS-KEY ( lymphoma* ) AND NOT TITLE-ABS-KEY ( "tumor* lys*" ) ) ) AND ( ( ( TITLE-ABS-KEY ( pharmacokinetic* ) OR TITLE-ABS-KEY ( ( drug* OR metabolite* ) W/2 ( level* OR concentration* ) ) OR TITLE-ABS-KEY ( ( medicine* OR drug* OR dos* ) W/2 ( precis* OR individuali?e* OR personali?e* OR monitor* ) ) OR TITLE-ABS-KEY ( ( pharmacokinetic* OR "non-linear mixed effect*" OR "nonlinear mixed effect*" OR pharmacometric* ) W/2 ( model* OR simulat* OR parameter* OR statistic* ) ) OR TITLE-ABS-KEY ( "*drug Monitor*" OR tdm* ) OR TITLE-ABS-KEY ( "dialy* clearance*" ) OR TITLE-ABS-KEY ( "population* pharmacokinetic*" ) OR TITLE-ABS-KEY ( ( population* ) W/2 ( pharmacokinetic* ) ) OR TITLE-ABS-KEY ( nlme* ) OR TITLE-ABS-KEY ( nonmem* OR "Nonlinear Mixed Effect* Model*" OR "Population Pharmacokinetic Model*" ) OR TITLE-ABS-KEY ( pharmacometric* ) OR TITLE-ABS-KEY ( ox?purinol* ) OR TITLE-ABS-KEY ( "Personali?ed dos*" OR "Individuali?ed dos*" OR "Precis* dos*" OR "Model-informed Precision Dos*" OR "Dos* Individuali?*" ) OR TITLE-ABS-KEY ( mipd* OR "dos* strateg*" ) ) ) OR ( ( TITLE-ABS-KEY ( pharmacodynamic* ) OR TITLE-ABS-KEY ( "dos* respon*" ) OR TITLE-ABS-KEY ( ( dos* OR drug* ) W/2 ( respon* OR eff?c* ) ) OR TITLE-ABS-KEY ( "Drug* eff?c*" ) OR TITLE-ABS-KEY ( "Treatment* Outcome*" OR "Therapeutic Index" OR "Treatment* Fail*" OR "Treatment* Terminat*" OR "Fatal Outcome*" ) OR TITLE-ABS-KEY ( "Disease Exacerbation*" ) OR TITLE-ABS-KEY ( "population* pharmacodynamic*" ) OR TITLE-ABS-KEY ( ( population* OR model* ) W/2 ( pharmacodynamic* ) ) OR TITLE-ABS-KEY ( "pharmacodynamic* model*" ) OR TITLE-ABS-KEY ( ( "uric acid*" OR urate* ) W/2 ( level* OR concentration* OR target* ) ) OR TITLE-ABS-KEY ( "uric acid*" ) OR TITLE-ABS-KEY ( urate* ) OR TITLE-ABS-KEY ( "PK*/PD*" ) OR TITLE-ABS-KEY ( "Pharmacokinetic*/pharmacodynamic*" ) OR TITLE-ABS-KEY ( ( "gout* flare*" OR "gout* attack*" ) W/2 ( frequen* OR number* OR mean* OR median* ) ) OR TITLE-ABS-KEY ( ( metabolite* OR drug* ) W/2 ( expos* ) ) OR TITLE-ABS-KEY ( ( "adverse event*" OR "adverse rection*" OR "side effect*" ) W/2 ( number* OR frequen* OR mean* OR median* ) ) ) ) ) |
| **All arms limited to human** |
| ( ( TITLE-ABS-KEY ( allopurinol* ) OR TITLE-ABS-KEY ( suppressant* AND gout* ) OR TITLE-ABS-KEY ( agent* AND antigout* ) OR TITLE-ABS-KEY ( antihyperuricemic* OR "Anti-hyperuricemic*" ) OR TITLE-ABS-KEY ( "urate* lower* therap*" OR "uric acid* lower* therap*" ) OR TITLE-ABS-KEY ( ( therap* OR treatment* ) W/2 ( "uric acid*" OR urate* ) ) OR TITLE-ABS-KEY ( "Xanthine oxidase inhibitor*" ) OR TITLE-ABS-KEY ( ( inhibit* OR deactivat* OR inactivat* ) W/2 ( "xanthine oxidase*" ) ) OR TITLE-ABS-KEY ( "Gout* Suppressant*" ) OR TITLE-ABS-KEY ( "Antigout* Agent*" ) OR TITLE-ABS-KEY ( zyloprim* ) OR TITLE-ABS-KEY ( lopurin* ) OR TITLE-ABS-KEY ( aloprim* ) OR TITLE-ABS-KEY ( zyloric* ) OR TITLE-ABS-KEY ( uricto* ) OR TITLE-ABS-KEY ( allosig ) OR TITLE-ABS-KEY ( progout* ) AND NOT TITLE-ABS-KEY ( rasburicase* ) ) ) AND ( ( TITLE-ABS-KEY ( "Home Dialys*" ) OR TITLE-ABS-KEY ( ( sorption* OR detoxification* ) W/2 ( blood* OR plasma* ) ) OR TITLE-ABS-KEY ( h*mofiltration* ) OR TITLE-ABS-KEY ( h*moperfusion* ) OR TITLE-ABS-KEY ( ( dialys* OR h*modialys* ) W/2 ( equipment* OR suppl* OR device* OR machine* OR catheter* OR membrane* OR center* OR patient* OR therap* OR solution* OR extracorporeal* ) ) OR TITLE-ABS-KEY ( ultrafiltration* ) OR TITLE-ABS-KEY ( h*modialys* ) OR TITLE-ABS-KEY ( dialys* ) OR TITLE-ABS-KEY ( ( dialys* ) W/2 ( kidney* OR renal* OR peritoneal* OR fluid* OR patient* OR "end stage renal disease*" ) ) OR TITLE-ABS-KEY ( *rrt* ) OR TITLE-ABS-KEY ( "renal replacement* therap*" ) AND NOT TITLE-ABS-KEY ( transplant* ) AND NOT TITLE-ABS-KEY ( leuk*mia* ) AND NOT TITLE-ABS-KEY ( lymphoma* ) AND NOT TITLE-ABS-KEY ( "tumor* lys*" ) ) ) AND ( ( ( TITLE-ABS-KEY ( pharmacokinetic* ) OR TITLE-ABS-KEY ( ( drug* OR metabolite* ) W/2 ( level* OR concentration* ) ) OR TITLE-ABS-KEY ( ( medicine* OR drug* OR dos* ) W/2 ( precis* OR individuali?e* OR personali?e* OR monitor* ) ) OR TITLE-ABS-KEY ( ( pharmacokinetic* OR "non-linear mixed effect*" OR "nonlinear mixed effect*" OR pharmacometric* ) W/2 ( model* OR simulat* OR parameter* OR statistic* ) ) OR TITLE-ABS-KEY ( "*drug Monitor*" OR tdm* ) OR TITLE-ABS-KEY ( "dialy* clearance*" ) OR TITLE-ABS-KEY ( "population* pharmacokinetic*" ) OR TITLE-ABS-KEY ( ( population* ) W/2 ( pharmacokinetic* ) ) OR TITLE-ABS-KEY ( nlme* ) OR TITLE-ABS-KEY ( nonmem* OR "Nonlinear Mixed Effect* Model*" OR "Population Pharmacokinetic Model*" ) OR TITLE-ABS-KEY ( pharmacometric* ) OR TITLE-ABS-KEY ( ox?purinol* ) OR TITLE-ABS-KEY ( "Personali?ed dos*" OR "Individuali?ed dos*" OR "Precis* dos*" OR "Model-informed Precision Dos*" OR "Dos* Individuali?*" ) OR TITLE-ABS-KEY ( mipd* OR "dos* strateg*" ) ) ) OR ( ( TITLE-ABS-KEY ( pharmacodynamic* ) OR TITLE-ABS-KEY ( "dos* respon*" ) OR TITLE-ABS-KEY ( ( dos* OR drug* ) W/2 ( respon* OR eff?c* ) ) OR TITLE-ABS-KEY ( "Drug* eff?c*" ) OR TITLE-ABS-KEY ( "Treatment* Outcome*" OR "Therapeutic Index" OR "Treatment* Fail*" OR "Treatment* Terminat*" OR "Fatal Outcome*" ) OR TITLE-ABS-KEY ( "Disease Exacerbation*" ) OR TITLE-ABS-KEY ( "population* pharmacodynamic*" ) OR TITLE-ABS-KEY ( ( population* OR model* ) W/2 ( pharmacodynamic* ) ) OR TITLE-ABS-KEY ( "pharmacodynamic* model*" ) OR TITLE-ABS-KEY ( ( "uric acid*" OR urate* ) W/2 ( level* OR concentration* OR target* ) ) OR TITLE-ABS-KEY ( "uric acid*" ) OR TITLE-ABS-KEY ( urate* ) OR TITLE-ABS-KEY ( "PK*/PD*" ) OR TITLE-ABS-KEY ( "Pharmacokinetic*/pharmacodynamic*" ) OR TITLE-ABS-KEY ( ( "gout* flare*" OR "gout* attack*" ) W/2 ( frequen* OR number* OR mean* OR median* ) ) OR TITLE-ABS-KEY ( ( metabolite* OR drug* ) W/2 ( expos* ) ) OR TITLE-ABS-KEY ( ( "adverse event*" OR "adverse rection*" OR "side effect*" ) W/2 ( number* OR frequen* OR mean* OR median* ) ) ) ) ) AND NOT ( ( INDEXTERMS ( animals OR animal ) ) AND NOT ( INDEXTERMS ( humans OR human ) ) ) |
| **All arms limited to human updated search 1 May 2024** |
| ( ( TITLE-ABS-KEY ( allopurinol* ) OR TITLE-ABS-KEY ( suppressant* AND gout* ) OR TITLE-ABS-KEY ( agent* AND antigout* ) OR TITLE-ABS-KEY ( antihyperuricemic* OR "anti-hyperuricemic*" ) OR TITLE-ABS-KEY ( "urate* lower* therap*" OR "uric acid* lower* therap*" ) OR TITLE-ABS-KEY ( ( therap* OR treatment* ) W/2 ( "uric acid*" OR urate* ) ) OR TITLE-ABS-KEY ( "xanthine oxidase inhibitor*" ) OR TITLE-ABS-KEY ( ( inhibit* OR deactivat* OR inactivat* ) W/2 ( "xanthine oxidase*" ) ) OR TITLE-ABS-KEY ( "gout* suppressant*" ) OR TITLE-ABS-KEY ( "antigout* agent*" ) OR TITLE-ABS-KEY ( zyloprim* ) OR TITLE-ABS-KEY ( lopurin* ) OR TITLE-ABS-KEY ( aloprim* ) OR TITLE-ABS-KEY ( zyloric* ) OR TITLE-ABS-KEY ( uricto* ) OR TITLE-ABS-KEY ( allosig ) OR TITLE-ABS-KEY ( progout* ) AND NOT TITLE-ABS-KEY ( rasburicase* ) ) ) AND ( ( TITLE-ABS-KEY ( "home dialys*" ) OR TITLE-ABS-KEY ( ( sorption* OR detoxification* ) W/2 ( blood* OR plasma* ) ) OR TITLE-ABS-KEY ( h*mofiltration* ) OR TITLE-ABS-KEY ( h*moperfusion* ) OR TITLE-ABS-KEY ( ( dialys* OR h*modialys* ) W/2 ( equipment* OR suppl* OR device* OR machine* OR catheter* OR membrane* OR center* OR patient* OR therap* OR solution* OR extracorporeal* ) ) OR TITLE-ABS-KEY ( ultrafiltration* ) OR TITLE-ABS-KEY ( h*modialys* ) OR TITLE-ABS-KEY ( dialys* ) OR TITLE-ABS-KEY ( ( dialys* ) W/2 ( kidney* OR renal* OR peritoneal* OR fluid* OR patient* OR "end stage renal disease*" ) ) OR TITLE-ABS-KEY ( *rrt* ) OR TITLE-ABS-KEY ( "renal replacement* therap*" ) AND NOT TITLE-ABS-KEY ( transplant* ) AND NOT TITLE-ABS-KEY ( leuk*mia* ) AND NOT TITLE-ABS-KEY ( lymphoma* ) AND NOT TITLE-ABS-KEY ( "tumor* lys*" ) ) ) AND ( ( ( TITLE-ABS-KEY ( pharmacokinetic* ) OR TITLE-ABS-KEY ( ( drug* OR metabolite* ) W/2 ( level* OR concentration* ) ) OR TITLE-ABS-KEY ( ( medicine* OR drug* OR dos* ) W/2 ( precis* OR individuali?e* OR personali?e* OR monitor* ) ) OR TITLE-ABS-KEY ( ( pharmacokinetic* OR "non-linear mixed effect*" OR "nonlinear mixed effect*" OR pharmacometric* ) W/2 ( model* OR simulat* OR parameter* OR statistic* ) ) OR TITLE-ABS-KEY ( "*drug monitor*" OR tdm* ) OR TITLE-ABS-KEY ( "dialy* clearance*" ) OR TITLE-ABS-KEY ( "population* pharmacokinetic*" ) OR TITLE-ABS-KEY ( ( population* ) W/2 ( pharmacokinetic* ) ) OR TITLE-ABS-KEY ( nlme* ) OR TITLE-ABS-KEY ( nonmem* OR "nonlinear mixed effect* model*" OR "population pharmacokinetic model*" ) OR TITLE-ABS-KEY ( pharmacometric* ) OR TITLE-ABS-KEY ( ox?purinol* ) OR TITLE-ABS-KEY ( "personali?ed dos*" OR "individuali?ed dos*" OR "precis* dos*" OR "model-informed precision dos*" OR "dos* individuali?*" ) OR TITLE-ABS-KEY ( mipd* OR "dos* strateg*" ) ) ) OR ( ( TITLE-ABS-KEY ( pharmacodynamic* ) OR TITLE-ABS-KEY ( "dos* respon*" ) OR TITLE-ABS-KEY ( ( dos* OR drug* ) W/2 ( respon* OR eff?c* ) ) OR TITLE-ABS-KEY ( "drug* eff?c*" ) OR TITLE-ABS-KEY ( "treatment* outcome*" OR "therapeutic index" OR "treatment* fail*" OR "treatment* terminat*" OR "fatal outcome*" ) OR TITLE-ABS-KEY ( "disease exacerbation*" ) OR TITLE-ABS-KEY ( "population* pharmacodynamic*" ) OR TITLE-ABS-KEY ( ( population* OR model* ) W/2 ( pharmacodynamic* ) ) OR TITLE-ABS-KEY ( "pharmacodynamic* model*" ) OR TITLE-ABS-KEY ( ( "uric acid*" OR urate* ) W/2 ( level* OR concentration* OR target* ) ) OR TITLE-ABS-KEY ( "uric acid*" ) OR TITLE-ABS-KEY ( urate* ) OR TITLE-ABS-KEY ( "pk*/pd*" ) OR TITLE-ABS-KEY ( "pharmacokinetic*/pharmacodynamic*" ) OR TITLE-ABS-KEY ( ( "gout* flare*" OR "gout* attack*" ) W/2 ( frequen* OR number* OR mean* OR median* ) ) OR TITLE-ABS-KEY ( ( metabolite* OR drug* ) W/2 ( expos* ) ) OR TITLE-ABS-KEY ( ( "adverse event*" OR "adverse rection*" OR "side effect*" ) W/2 ( number* OR frequen* OR mean* OR median* ) ) ) ) ) AND NOT ( ( INDEXTERMS ( animals OR animal ) ) AND NOT ( INDEXTERMS ( humans OR human ) ) ) AND ( LIMIT-TO ( PUBYEAR , 2023 ) OR LIMIT-TO ( PUBYEAR , 2024 ) ) |

### **Web of Science**

| **#** | **Web of Science Core Collection database queries** |
| --- | --- |
|  | Search: TS=(Progout*) |
|  | Search: TS=(Allosig) |
|  | Search: TS=(Uricto*) |
|  | Search: TS=(Zyloric*) |
|  | Search: TS=(Aloprim*) |
|  | Search: TS=(Lopurin*) |
|  | Search: TS=(Zyloprim*) |
|  | Search: TS=("Antigout* Agent*" OR "Anti-gout* Agent*") |
|  | Search: TS=("Gout* Suppressant*") |
|  | Search: TS=((inhibit* OR deactivat* OR inactivat*) NEAR/3 ("xanthine oxidase*")) |
|  | Search: TS=("Xanthine oxidase inhibitor*") |
|  | Search: TS=((therap* OR treatment*) NEAR/3 ("uric acid*" OR urate*)) |
|  | Search: TS=("urate* lower* therap*" OR "urate*-lower* therap*" OR "uric acid* lower* therap*" OR "uric acid*-lower* therap*") |
|  | Search: TS=(Antihyperuricemic* OR "Anti-hyperuricemic*") |
|  | Search: TS=(Agent* AND Antigout*) |
|  | Search: TS=(suppressant* AND gout*) |
|  | Search: TS=(Allopurinol*) |
|  | Search: (#1 OR #2 OR #3 OR #4 OR #5 OR #6 OR #7 OR #8 OR #9 OR #10 OR #11 OR #12 OR #13 OR #14 OR #15 OR #16 OR #17) not (TS= rasburicase) |
|  | Search: TS=("Home Dialys*") |
|  | Search: TS=((Sorption* OR Detoxification*) NEAR/3 (blood* OR plasma*)) |
|  | Search: TS=(Hemofiltration*) |
|  | Search: TS=(Hemoperfusion*) |
|  | Search: TS=((Dialys* OR he$modialys*) NEAR/3 (Equipment* OR suppl* OR device* OR machine* OR catheter* OR membrane* OR center* OR patient* OR therap* OR solution* OR Extracorporeal*)) |
|  | Search: TS=(Ultrafiltration*) |
|  | Search: TS=(He$modialys*) |
|  | Search: TS=(Dialys*) |
|  | Search: TS=((dialys*) NEAR/3 (kidney* OR renal* OR peritoneal* OR fluid* OR "end stage renal disease*" OR "end-stage renal disease*")) |
|  | Search: TS=("renal replacement* therap*") and Crrt (Should – Search within topic) and Renal Replacement Therapy Rrt (Should – Search within topic) |
|  | Search: (#19 OR #20 OR #21 OR #22 OR #23 OR #24 OR #25 OR #26 OR #27 OR #28) not (TS= transplant*) not (TS= leuk$emia*) not (TS= lymphoma*) not (TS= "tumor* lys*") |
|  | Search: TS=(pharmacokinetic*) |
|  | Search: TS=((drug* OR metabolite*) NEAR/3 (level* or concentration*)) |
|  | Search: TS=((medicine* OR drug* OR dos*) NEAR/3 (precis* OR individuali?e* OR personali?e* OR monitor*)) |
|  | Search: TS=((pharmacokinetic* OR "non-linear mixed effect*" OR "nonlinear mixed effect*" OR pharmacometric*) NEAR/3 (Model* OR simulat* OR parameter* OR statistic*)) |
|  | Search: TS=("Drug Monitor*") and Therapeutic Drug Monitoring (Should – Search within topic) and Tdm (Should – Search within topic) and Prescription Drug Monitoring Program (Should – Search within topic) and Pharmacokinetics (Should – Search within topic) and Population Pharmacokinetics (Should – Search within topic) |
|  | Search: TS=("dialy* clearance*") |
|  | Search: TS=("population* pharmacokinetic*") |
|  | Search: TS=((Population*) NEAR/3 (pharmacokinetic*)) |
|  | Search: TS=(NLME*) |
|  | Search: TS=(NONMEM*) and Nonmem (Should – Search within topic) and Population Pharmacokinetic (Should – Search within topic) and Nonlinear Mixed Effects Modeling (Should – Search within topic) and Population Pharmacokinetic Model (Should – Search within topic) and Pharmacometrics (Should – Search within topic) |
|  | Search: TS=(Ox?purinol*) |
|  | Search: TS=("Personali?ed dos*" OR "Individuali?ed dos*" OR "Precis* dos*") and Model-informed Precision Dosing (Should – Search within topic) and Individualized Dosing (Should – Search within topic) and Precision Dosing (Should – Search within topic) and Dose Individualisation (Should – Search within topic) and Individualized Dosage (Should – Search within topic) |
|  | Search: TS=(MIPD* OR "dos* strateg*") and Dosing Strategy (Should – Search within topic) and Dosing Strategies (Should – Search within topic) and Mipd (Should – Search within topic) and Dosage Strategy (Should – Search within topic) |
|  | Search: #30 OR #31 OR #32 OR #33 OR #34 OR #35 OR #36 OR #37 OR #38 OR #39 OR #40 OR #41 OR #42 |
|  | Search: TS=(pharmacodynamic*) and Pharmacodynamics (Should – Search within topic) and Pharmacodynamic (Should – Search within topic) and Pharmacokinetics Pharmacodynamics (Should – Search within topic) and Pk Pd (Should – Search within topic) and Pharmacokinetics-pharmacodynamics (Should – Search within topic) |
|  | Search: TS=("dos* respon*") and Dose-response (Should – Search within topic) and Dose Response (Should – Search within topic) and Dose-response Relationship (Should – Search within topic) and Dose-response Curve (Should – Search within topic) and Dose-response Analysis (Should – Search within topic) and Dose-response Model (Should – Search within topic) and Dose-response Meta-analysis (Should – Search within topic) and Dose-response Curves (Should – Search within topic) |
|  | Search: TS=((Dos* OR drug*) NEAR/3 (respon* OR eff?c*)) |
|  | Search: TS=("Drug* eff?c*") and Drug Effects (Should – Search within topic) and Drug Efficacy (Should – Search within topic) and Adverse Drug Effects (Should – Search within topic) and Adverse Drug Effect (Should – Search within topic) and Drug Effectiveness (Should – Search within topic) and Drug Toxicity Drug Effects (Should – Search within topic) |
|  | Search: TS=("Treatment* Outcome*" OR "Therapeutic Index" OR "Treatment* Fail*" OR "Treatment* Terminat*" OR "Fatal Outcome*") and Treatment Outcome (Should – Search within topic) and Treatment Outcomes (Should – Search within topic) and Treatment Failure (Should – Search within topic) and Fatal Outcome (Should – Search within topic) and Therapeutic Index (Should – Search within topic) and Prediction Of Treatment Outcome (Should – Search within topic) and Treatment Outcome Prediction (Should – Search within topic) |
|  | Search: TS=("Disease Exacerbation*") |
|  | Search: TS=("population* pharmacodynamic*") and Population Pharmacodynamics (Should – Search within topic) and Pharmacodynamic Model (Should – Search within topic) |
|  | Search: TS=((population* OR model*) NEAR/3 (pharmacodynamic*)) |
|  | Search: TS=("pharmacodynamic* model*") |
|  | Search: TS=(("uric acid*"OR urate*) NEAR/3 (level* OR concentration* OR target*)) |
|  | Search: TS=(("uric acid*"OR urate*) NEAR/3 (level* OR concentration* OR target*)) and Uric Acid (Should – Search within topic) and Serum Uric Acid (Should – Search within topic) and Urate (Should – Search within topic) and Serum Urate (Should – Search within topic) |
|  | Search: TS=("uric acid*") |
|  | Search: TS=(urate*) |
|  | Search: TS=("PK*/PD*") and Pk Pd (Should – Search within topic) and Pk Pd Modeling (Should – Search within topic) and Pk-pd (Should – Search within topic) and Pk-pd Modeling (Should – Search within topic) and Pk Pd Model (Should – Search within topic) and Pk Pd Modelling (Should – Search within topic) and Pk-pd Model (Should – Search within topic) and Pharmacokinetics Pharmacodynamics (Should – Search within topic) |
|  | Search: TS=("Pharmacokinetic*/pharmacodynamic*") |
|  | Search: TS=(("gout* flare*" OR "gout* attack*") NEAR/3 (frequen* OR number* OR mean* OR median*)) and Gout Flares (Should – Search within topic) and Gout Flare (Should – Search within topic) |
|  | Search: TS=((metabolite* OR drug*) NEAR/3 (expos*)) and Drug Exposure (Should – Search within topic) |
|  | Search: TS=(("adverse event*" OR "adverse rection*" OR "side effect*") NEAR/3 (number* OR frequen* OR mean* OR median*)) |
|  | Search: #44 OR #45 OR #46 OR #47 OR #48 OR #49 OR #50 OR #51 OR #52 OR #53 OR #54 OR #55 OR #56 OR #57 OR #58 OR #59 OR #60 OR #61 |
|  | Search: #43 OR #62 |
|  | Search: #18 AND #29 AND #63 |

### **CINAHL**

| **#** | **Query** |
| --- | --- |
| S72 | S14 AND S28 AND S70^ab^ |
| S71 | S14 AND S28 AND S70^bc^ |
| S70 | S48 OR S69^bd^ |
| S69 | S49 OR S50 OR S51 OR S52 OR S53 OR S54 OR S55 OR S56 OR S57 OR S58 OR S59 OR S60 OR S61 OR S62 OR S63 OR S64 OR S65 OR S66 OR S67 OR S68 ^bd^ |
| S68 | TX ("adverse event*" OR "adverse rection*" OR "side effect*") N3 (number* OR frequen* OR mean* OR median*) ^bd^ |
| S67 | TX (metabolite* OR drug*) N3 (expos*) ^bd^ |
| S66 | TX ("gout* flare*" OR "gout* attack*")  N3 (frequen* OR number* OR mean* OR median*) ^bd^ |
| S65 | TX "Pharmacokinetic*/pharmacodynamic*" ^bd^ |
| S64 | TX "PK*/PD*" ^bd^ |
| S63 | TX urate* ^bd^ |
| S62 | TX "uric acid*" ^bd^ |
| S61 | TX (uric acid* OR urate*) N3 (level* OR concentration* OR target*) ^bd^ |
| S60 | TX "pharmacodynamic* model*" ^bd^ |
| S59 | TX (population* OR model*) N3 (pharmacodynamic*) ^bd^ |
| S58 | TX "population* pharmacodynamic*" ^bd^ |
| S57 | (MH "Disease Exacerbation") ^bd^ |
| S56 | (MH "Treatment Outcomes") OR (MH "Drug Efficacy") OR (MH "Therapeutic Index") OR (MH "Treatment Failure") OR (MH "Treatment Termination") OR (MH "Fatal Outcome") ^bd^ |
| S55 | (MM "Adverse Drug Event") ^bd^ |
| S54 | TX "dos* respon*" ^bd^ |
| S53 | TX "Drug* eff?c*" ^bd^ |
| S52 | TX (Dos* OR drug*) N3 (respon* OR eff?c*) ^bd^ |
| S51 | (MH "Dose-Response Relationship, Drug") ^bd^ |
| S50 | (MH "Uric Acid") ^bd^ |
| S49 | TX pharmacodynamic * ^bd^ |
| S48 | S29 OR S30 OR S31 OR S32 OR S33 OR S34 OR S35 OR S36 OR S37 OR S38 OR S39 OR S40 OR S41 OR S42 OR S43 OR S44 OR S45 OR S46 OR S47 ^bd^ |
| S47 | TX (Dos*) N3 (Personali?ed OR Individuali?ed OR Precis*) ^bd^ |
| S46 | TX "Precis* dos*" ^bd^ |
| S45 | TX "Individuali?ed dos*" ^bd^ |
| S44 | TX "Personali?ed dos*" ^bd^ |
| S43 | TX Ox?purinol* ^bd^ |
| S42 | TX NONMEM* ^bd^ |
| S41 | TX NLME* ^bd^ |
| S40 | TX (Population*) N3 (pharmacokinetic*) ^bd^ |
| S39 | TX "population* pharmacokinetic*" ^bd^ |
| S38 | TX (Drug*) N3 (level* or concentration*) ^bd^ |
| S37 | TX "dialy* clearance*" ^bd^ |
| S36 | (MH "Drug Monitoring") ^bd^ |
| S35 | TX (pharmacokinetic* OR "non-linear mixed effect*" OR "nonlinear mixed effect*" OR pharmacometric*) N3 (Model* OR simulat* OR parameter*) ^bd^ |
| S34 | (MH "Models, Statistical+") ^bd^ |
| S33 | (MH "Individualized Medicine") ^bd^ |
| S32 | (MH "Drug Evaluation+") ^bd^ |
| S31 | (MH "Osmolar Concentration+") ^bd^ |
| S30 | TX pharmacokinetic* ^bd^ |
| S29 | (MH "Pharmacokinetics+") ^bd^ |
| S28 | (S15 OR S16 OR S17 OR S18 OR S19 OR S20 OR S21 OR S22 OR S23 OR S24 OR S25 OR S26 OR S27) NOT (TX transplant*) NOT (TX leuk#emia*) NOT (TX lymphoma*) NOT (TX "tumor* lys*") ^bd^ |
| S27 | TX "renal replacement* therap*" ^bd^ |
| S26 | TX (dialys*) N3 (kidney* OR renal* OR peritoneal* OR fluid* OR patient* OR "end stage renal disease*" OR "end- stage renal disease*") ^bd^ |
| S25 | TX Dialys* ^bd^ |
| S24 | TX he#modialys * ^bd^ |
| S23 | (MM "Extracorporeal Circulation") OR (MH "Ultrafiltration+") ^bd^ |
| S22 | (MH "Dialysis Solutions") ^bd^ |
| S21 | (MH "Hemodialysis Therapy (Iowa NIC)") OR (MH "Peritoneal Dialysis Therapy (Iowa NIC)") ^bd^ |
| S20 | (MH "Dialysis Patients") ^bd^ |
| S19 | (MH "Dialysis Centers") ^bd^ |
| S18 | (MH "Dialysis Equipment and Supplies+") ^bd^ |
| S17 | (MM "Renal Replacement Therapy") OR (MH "Continuous Renal Replacement Therapy+") ^bd^ |
| S16 | (MM "Sorption Detoxification") OR (MH "Hemodialysis+") OR (MH "Hemofiltration+") OR (MH "Hemoperfusion") ^bd^ |
| S15 | (MH "Dialysis") OR (MH "Hemodialysis") OR (MH "Home Dialysis") OR (MH "Peritoneal Dialysis+") ^bd^ |
| S14 | (S1 OR S2 OR S3 OR S4 OR S5 OR S6 OR S7 OR S8 OR S9 OR S10 OR S11 OR S12 OR S13) NOT (TX rasburicase*) ^bd^ |
| S13 | TX Lopurin* ^bd^ |
| S12 | TX Zyloprim* ^bd^ |
| S11 | TX "Antigout* Agent*" ^bd^ |
| S10 | TX "Gout* Suppressant*" ^bd^ |
| S9 | TX (inhibit* OR deactivat* OR inactivat*) N3 ("xanthine oxidase*") ^bd^ |
| S8 | TX "Xanthine oxidase inhibitor*" ^bd^ |
| S7 | TX (therap* OR treatment*) N3 ("uric acid*" OR urate*) ^bd^ |
| S6 | TX ("urate* lower* therap*" OR "uric acid* lower* therap*") ^bd^ |
| S5 | TX (Antihyperuricemic* OR "Anti- hyperuricemic*") ^bd^ |
| S4 | TX (Agent* AND Antigout*) ^bd^ |
| S3 | TX (suppressant* AND gout*) ^bd^ |
| S2 | TX Allopurinol* ^bd^ |
| S1 | MM Enzyme Inhibitors OR MH Allopurinol OR MM Antimetabolites OR MM Antirheumatic Agents OR MM Gout Suppressants OR MM Antimetabolites, Antineoplastic ^bd^ |

^a^: Limiters - Publication Date: 20231001-20240531; Human, Expanders – Apply equivalent subjects Search modes - Boolean/Phrase; ^b^: Last Run via Interface – EBSCOhost Research Databases Search Screen - Advanced Search Database - CINAHL Complete; ^c^: Limiters – Human Expanders - Apply equivalent subjects Search modes -Boolean/Phrase; ^d^: Expanders - Apply equivalent subjects Search modes - Boolean/Phrase

### **Medline**

| **#** | **Query** |
| --- | --- |
| 1 | Allopurinol/ |
| 2 | Allopurinol*.tw,kf. |
| 3 | (suppressant* and gout*).tw,kf. |
| 4 | gout suppressants/ or exp allopurinol/ |
| 5 | (Agent* and Antigout*).tw,kf. |
| 6 | Antigout* Agent*.tw,kf. |
| 7 | (Antihyperuricemic* or Anti-hyperuricemic*).tw,kf. |
| 8 | (urate* lower* therap* or uric acid* lower* therap*).tw,kf. |
| 9 | ((therap* or treatment*) adj3 (uric acid* or urate*)).tw,kf. |
| 10 | Xanthine oxidase inhibitor*.tw,kf. |
| 11 | ((inhibit* or deactivat* or inactivat*) adj3 xanthine oxidase*).tw,kf. |
| 12 | Gout* Suppressant*.tw,kf. |
| 13 | Antigout* Agent*.tw,kf. |
| 14 | Zyloprim*.tw,kf. |
| 15 | Lopurin*.tw,kf. |
| 16 | Aloprim*.tw,kf. |
| 17 | (1 or 2 or 3 or 4 or 5 or 6 or 7 or 8 or 9 or 10 or 11 or 12 or 13 or 14 or 15 or 16) not rasburicase*.tw,kf. |
| 18 | Dialysis/ |
| 19 | renal replacement therapy/ or continuous renal replacement therapy/ or hemofiltration/ or hemoperfusion/ or hybrid renal replacement therapy/ or intermittent renal replacement therapy/ or exp renal dialysis/ or exp peritoneal dialysis/ |
| 20 | exp Dialysis Solutions/ |
| 21 | he?modialys*.tw,kf. |
| 22 | Dialys*.tw,kf. |
| 23 | (dialys* adj3 (kidney* or renal* or peritoneal* or fluid* or patient* or end stage renal disease* or end-stage renal disease*)).tw,kf. |
| 24 | renal replacement* therap*.tw,kf. |
| 25 | (18 or 19 or 20 or 21 or 22 or 23 or 24) not transplant*.tw,kf. not leuk*emia*.tw,kf. not lymphoma*.tw,kf. not tumor* lys*.tw,kf. |
| 26 | exp Pharmacokinetics/ |
| 27 | pharmacokinetic*.tw,kf. |
| 28 | ((pharmacokinetic* or non-linear mixed effect* or nonlinear mixed effect* or pharmacometric*) adj3 (Model* or simulat* or parameter*)).tw,kf. |
| 29 | models, statistical/ or exp likelihood functions/ or exp linear models/ or exp nomograms/ or exp monte carlo method/ or probability/ or exp bayes theorem/ or exp markov chains/ or exp uncertainty/ or regression analysis/ or exp least-squares analysis/ or exp spatial regression/ or exp statistical distributions/ or exp statistics, nonparametric/ or exp stochastic processes/ |
| 30 | dialy* clearance*.tw,kf. |
| 31 | (Drug* adj3 (level* or concentration*)).tw,kf. |
| 32 | Population* pharmacokinetic*.tw,kf. |
| 33 | (Population* adj3 pharmacokinetic*).tw,kf. |
| 34 | NLME*.tw,kf. |
| 35 | NONMEM*.tw,kf. |
| 36 | Ox#purinol*.tw,kf. |
| 37 | Personali#ed dos*.tw,kf. |
| 38 | Individuali#ed dos*.tw,kf. |
| 39 | Precis* dos*.tw,kf. |
| 40 | (Dos* adj3 (Personali#ed or Individuali#ed or Precis*)).tw,kf. |
| 41 | 26 or 27 or 28 or 29 or 30 or 31 or 32 or 33 or 34 or 35 or 36 or 37 or 38 or 39 or 40 |
| 42 | pharmacodynamic*.tw,kf. |
| 43 | exp Uric Acid/ |
| 44 | Uric acid*.tw,kf. |
| 45 | Urate*.tw,kf. |
| 46 | exp Dose-Response Relationship, Drug/ |
| 47 | dos* respon*.tw,kf. |
| 48 | ((Dos* or drug*) adj3 (respon* or eff#c*)).tw,kf. |
| 49 | Drug* eff#c*.tw,kf. |
| 50 | Population* pharmacodynamic*.tw,kf. |
| 51 | ((Population* or model*) adj3 pharmacodynamic*).tw,kf. |
| 52 | pharmacodynamic* model*.tw,kf. |
| 53 | ((uric acid* or urate*) adj3 (level* or concentration* or target*)).tw,kf. |
| 54 | Uric acid*.tw,kf. |
| 55 | Urate*.tw,kf. |
| 56 | "PK*/PD*".tw,kf. |
| 57 | "pharmacokinetic*/pharmacodynamic*".tw,kf. |
| 58 | ((metabolite* or drug*) adj3 expos*).tw,kf. |
| 59 | ((Gout* flare* or gout* attack*) adj3 (frequen* or number* or mean* or median*)).tw,kf. |
| 60 | ((adverse event* or adverse rection* or side effect*) adj3 (number* or frequen* or mean* or median*)).tw,kf. |
| 61 | 42 or 43 or 44 or 45 or 46 or 47 or 48 or 49 or 50 or 51 or 52 or 53 or 54 or 55 or 56 or 57 or 58 or 59 or 60 |
| 62 | 41 or 61 |
| 63 | 17 and 25 and 62 |
| 64 | exp symptom flare up/ |
| 65 | "drug-related side effects and adverse reactions"/ or drug hypersensitivity/ or exp drug eruptions/ or exp drug fever/ |
| 66 | 42 or 43 or 44 or 45 or 46 or 47 or 48 or 49 or 50 or 51 or 52 or 53 or 54 or 55 or 56 or 57 or 58 or 59 or 60 or 61 or 62 or 63 or 64 or 65 |
| 67 | 41 or 66 |
| 68 | 17 and 25 and 67 |
| 69 | limit 68 to humans |
| 70 | limit 69 to dt="20231019-20240501" |

## **Identified clinical trials**

Two studies were relevant but excluded from the systematic review analysis because they were clinical trial protocols with no published results. The first was a pilot study in peritoneal dialysis patients with gout on allopurinol with the aim to investigate oxypurinol pharmacokinetics (ACTRN12620001005943). The authors excluded patients on automated peritoneal dialysis or with peritonitis. The second study was in haemodialysis gout patients. As a chronotherapy study, the intervention was to change the time of allopurinol administration to bedtime (post-dialysis) instead of pre-dialysis at the same dose. The primary outcome was to compare the efficacy of allopurinol in serum urate lowering upon changing the time of its administration (NCT02477488). Details of the search conducted in clinical trial registries and the relevant trials are presented below.

### **Clinical trials.gov**

Condition or disease: Renal Dialysis. Intervention/Treatment: Allopurinol

Results: 2 (the ALTERED trial (NCT01951404) irrelevant due to exclusion of gout patients. The other trial was relevant and detailed below):

| NCT Number | NCT02477488 |
| --- | --- |
| Study Title | Optimal Administration of Allopurinol in Dialysis Patients: A Chronotherapy Trial |
| Study Status | COMPLETED (2015-2016) |
| Study results | No results posted |

### **WHO ICTRP**

Four trials found upon searching for: dialysis AND Allopurinol: ACTRN12620001005943, IRCT20090905002417N20, NCT02477488 , ACTRN12614000328673, among which the same previous relevant chronotherapy trial and another relevant trial in peritoneal dialysis detailed below:

| Trial ID | ACTRN12620001005943 |
| --- | --- |
| Study Title | A pilot study to determine the impact of peritoneal dialysis on oxypurinol and urate handling in patients with gout |
| Study Status | Recruitment completed (2020-2022) |
| Study results | Published in 2024, Wilson *et al.*’s study [1] |

### **CENTRAL**

45 **Trials matching** the search *Renal dialysis AND Allopurinol in All Text, no new trials other than the previous two were relevant.

## **References**

1. Wilson LC, Ward J, Wright DFB, Green SC, Stocker SL, Putt TL, Schollum JBW, et al (2024) *The impact of peritoneal dialysis on oxypurinol and urate elimination in people with gout.* Nephrology (Carlton). **29**:547-550. <https://doi.org/10.1111/nep.14306>
